# Prenatal Alcohol Exposure: Profiling Developmental DNA Methylation Patterns in Central and Peripheral Tissues

**DOI:** 10.3389/fgene.2018.00610

**Published:** 2018-12-04

**Authors:** Alexandre A. Lussier, Tamara S. Bodnar, Matthew Mingay, Alexandre M. Morin, Martin Hirst, Michael S. Kobor, Joanne Weinberg

**Affiliations:** ^1^Department of Cellular & Physiological Sciences, Faculty of Medicine, Life Sciences Institute, University of British Columbia, Vancouver, BC, Canada; ^2^Centre for Molecular Medicine and Therapeutics, Department of Medical Genetics, British Columbia Children's Hospital Research Institute, University of British Columbia, Vancouver, BC, Canada; ^3^Department of Microbiology and Immunology, Michael Smith Laboratories Centre for High-Throughput Biology, University of British Columbia, Vancouver, BC, Canada; ^4^Canada's Michael Smith Genome Sciences Centre, BC Cancer Agency Research Centre, BC Cancer Agency, Vancouver, BC, Canada; ^5^Human Early Learning Partnership, University of British Columbia, Vancouver, BC, Canada

**Keywords:** prenatal alcohol exposure, fetal alcohol spectrum disorder, brain, development, epigenetics, DNA methylation, immune

## Abstract

**Background:** Prenatal alcohol exposure (PAE) can alter the development of neurobiological systems, leading to lasting neuroendocrine, neuroimmune, and neurobehavioral deficits. Although the etiology of this reprogramming remains unknown, emerging evidence suggests DNA methylation as a potential mediator and biomarker for the effects of PAE due to its responsiveness to environmental cues and relative stability over time. Here, we utilized a rat model of PAE to examine the DNA methylation profiles of rat hypothalami and leukocytes at four time points during early development to assess the genome-wide impact of PAE on the epigenome and identify potential biomarkers of PAE. Our model of PAE resulted in blood alcohol levels of ~80–150 mg/dl throughout the equivalent of the first two trimesters of human pregnancy. Hypothalami were analyzed on postnatal days (P) 1, 8, 15, 22 and leukocytes at P22 to compare central and peripheral markers. Genome-wide DNA methylation analysis was performed by methylated DNA immunoprecipitation followed by next-generation sequencing.

**Results:** PAE resulted in lasting changes to DNA methylation profiles across all four ages, with 118 differentially methylated regions (DMRs) displaying persistent alterations across the developmental period at a false-discovery rate (FDR) < 0.05. In addition, 299 DMRs showed the same direction of change in the hypothalamus and leukocytes of P22 pups at an FDR < 0.05, with some genes overlapping with the developmental profile findings. The majority of these DMRs were located in intergenic regions, which contained several computationally-predicted transcription factor binding sites. Differentially methylated genes were generally involved in immune function, epigenetic remodeling, metabolism, and hormonal signaling, as determined by gene ontology analyses.

**Conclusions:** Persistent DNA methylation changes in the hypothalamus may be associated with the long-term physiological and neurobehavioral alterations in observed in PAE. Furthermore, correlations between epigenetic alterations in peripheral tissues and those in the brain will provide a foundation for the development of biomarkers of fetal alcohol spectrum disorder (FASD). Finally, findings from studies of PAE provide important insight into the etiology of neurodevelopmental and mental health disorders, as they share numerous phenotypes and comorbidities.

## Introduction

Early-life environments influence the development of biological/neurobiological systems, leading to long-term consequences in offspring (Godfrey and Robinson, 1998; Hanson and Gluckman, 2008). In particular, prenatal alcohol exposure (PAE) can result in Fetal Alcohol Spectrum Disorders (FASD) in humans, which are associated with a wide variety of adverse effects. Exposure to alcohol at high levels throughout pregnancy can result in Fetal Alcohol Syndrome (FAS), characterized by growth retardation, a characteristic facial dysmorphology, and multiple central nervous system alterations. Exposure to alcohol at levels that do not produce FAS can result in either partial FAS (pFAS), where only some of the diagnostic features are observed, or in numerous alcohol-related neurobehavioral effects (alcohol-related neurodevelopmental disorder, ARND; Stratton et al., 1996). The degree to which alcohol affects development depends on a variety of factors such as timing, pattern, and level of alcohol exposure, overall maternal health and nutrition, and genetic background (Pollard, 2007), which may influence the broad range of effects of *in utero* alcohol exposure and the relatively high prevalence of FASD (Riley et al., 2011; May et al., 2018). Importantly, PAE can alter the development, function, and regulation of numerous neurobiological and physiological systems, giving rise to lasting deficits across the spectrum of FASD, including, but not limited to cognitive and behavioral deficits, impairment to self-regulation and adaptive functioning, immune dysregulation, and increased vulnerability to mental health problems across the lifespan (Zhang et al., 2005; Mattson et al., 2011; Pei et al., 2011).

The hypothalamus is highly susceptible to the programming effects of PAE (Eguchi, 1969; Matthews, 2002; Hellemans et al., 2008; Weinberg et al., 2008). The hypothalamus plays key roles in neuroendocrine regulation, autonomic regulation, and homeostatic control, regulating growth, sleep/wake behavior, circadian rhythms, metabolism, body temperature, and other vital functions (Card and Swanson, 2012). Data from both human clinical cohorts and animal models of FASD have identified alterations to physiological functions associated with the hypothalamus. For example, infants exposed to alcohol *in utero* show both elevated basal and post-stress levels of cortisol, and children with FASD who experience early life adversity exhibit dysregulation of the cortisol circadian rhythm (Keiver et al., 2015; McLachlan et al., 2016). Similarly, in animal models of PAE, exposed offspring exhibit hyperresponsiveness to stressors as well as altered central regulation of hypothalamic-pituitary-adrenal (HPA) axis activity (Ramsay et al., 1996; Jacobson et al., 1999; Haley et al., 2006; Weinberg et al., 2008). Furthermore, PAE also alters sleep patterns and circadian rhythms, results in deficits in thermoregulation, and is associated with inappropriate feeding behavior (Jones and Smith, 1973; Zimmerberg et al., 1987; Earnest et al., 2001; Sei et al., 2003; Chen et al., 2012; Werts et al., 2014). These deficits often persist across the life course, suggesting that alcohol exposure during prenatal life may alter developmental trajectories to increase the risk of adverse outcomes (Hellemans et al., 2010a). In the context of the fetal programming hypothesis, early environmental or non-genetic factors, including maternal undernutrition, stress, and exposure to drugs or other toxic agents, can permanently organize or imprint physiological and neurobiological systems and increase adverse cognitive, adaptive, and behavioral outcomes, as well as vulnerability to diseases or disorders later in life (Godfrey and Robinson, 1998; Hanson and Gluckman, 2008; Swanson et al., 2009). As the underlying mechanisms of these effects begin to emerge, it has become apparent that epigenetic mechanisms might be important candidates for the programming effects of PAE, linking early life environmental factors and neurobiological outcomes while influencing health and behavior well into adulthood (Yuen et al., 2011; Shulha et al., 2013). The term epigenetics broadly refers to the modifications of DNA and its packaging that alter DNA accessibility, which modulates gene expression and cell functions without changes to underlying genomic sequences (Bird, 2007). These epigenetic factors include direct modifications to DNA, post-translational modification of histones, and non-coding RNAs.

DNA methylation currently is the most studied epigenetic modification and involves the covalent attachment of a methyl group to the 5′ position of cytosine, typically occurring at cytosine-guanine dinucleotide (CpG) sites (Jones and Takai, 2001). Although closely linked to the regulation of gene expression, the association between DNA methylation and transcription depends on genomic context. Whereas, DNA methylation typically represses gene expression when located in promoter regions, its effects are more variable for CpGs residing in gene bodies and intergenic regions. DNA methylation can also directly control transcription factor binding to gene regulatory regions, such as enhancers, to modulate gene expression patterns (Tate and Bird, 1993). In addition to this role in transcriptional control, DNA methylation has been associated with altered mRNA splicing when located within introns, and its presence within certain exons may potentially regulate alternative transcriptional start sites (Maunakea et al., 2010, 2013; Shukla et al., 2011). Furthermore, DNA methylation is closely linked to several important developmental processes, including genomic imprinting, tissue specification, and differentiation, suggesting a role in the regulation of cellular functions and developmental trajectories (Smith and Meissner, 2013; Ziller et al., 2014). Perhaps most importantly, DNA methylation can be responsive to environmental influences and these changes can possibly be inherited through cell divisions to potentially persist throughout life (Hanson et al., 2011; Langevin et al., 2011; Yuen et al., 2011). An additional interesting aspect of DNA methylation is its emerging role as a potential biomarker of early-life exposures, as it is easily quantifiable, stable over time, and can be obtained from readily available peripheral tissues, such as buccal epithelial cells and white blood cells (Bock, 2009).

Given its role in the regulation of gene expression and cell function, as well as its responsiveness to environmental factors, DNA methylation provides an attractive mechanism for the biological embedding of the persistent deficits caused by PAE. Mounting evidence suggests a potential role in the etiology of PAE-induced deficits, as numerous studies have identified alterations to epigenetic programs in the central nervous system of animals exposed to alcohol *in utero* across various levels and stages of exposure (reviewed in Lussier et al., 2017). These range from differences in bulk levels of DNA methylation to genome-wide changes in DNA methylation patterns, supporting the hypothesis that PAE can alter the epigenome (Bekdash et al., 2013; Laufer et al., 2013). Although genome-wide studies have been performed on whole brains in mice, few studies have focused on targeted brain regions. Among those that have, PAE was shown to be associated with altered DNA methylation status of the *POMC* promoter in the rat hypothalamus (Bekdash et al., 2013; Ngai et al., 2015). As a key regulator of the stress response, alterations to this gene may reflect broader alterations to the regulatory functions of the hypothalamus. Studies from clinical cohorts of children with FASD have identified widespread changes to DNA methylation patterns in peripheral tissues (Laufer et al., 2015; Portales-Casamar et al., 2016; Lussier et al., 2018). However, alterations to central tissue are difficult if not impossible to directly assess in clinical populations, and while peripheral tissues are more easily accessible, changes in these cells may not fully reflect alterations in the brain (Berko et al., 2014). Furthermore, biological embedding of PAE's effects earlier in development could potentially lead to more systemic effects on the epigenome, which would be reflected by alterations present across a variety of tissues.

Currently, the genome-wide impact of PAE on DNA methylation within the hypothalamus remains unknown (Bekdash et al., 2013; Ngai et al., 2015). To address this gap, we utilized a well-established rat model of PAE to assess whether moderate to moderately high levels of first and second trimester-equivalent alcohol exposure alters DNA methylation profiles in the early postnatal period, and whether altered sites of DNA methylation could serve as biomarkers of gestational alcohol exposure if also identified in peripheral tissues. Using methylated DNA immunoprecipitation and next-generation sequencing (meDIP-seq), we identified statistically significant differentially methylated regions (DMRs) in female PAE animals that persisted across preweaning development of the hypothalamus. In parallel, we identified concordant DNA methylation alterations in white blood cells and the hypothalamus of female PAE animals compared to controls on postnatal day (P) 22. Our findings suggest that: (1) PAE alters DNA methylation patterns in both central and peripheral tissues, potentially reprogramming neurobiological/physiological systems and influencing the deficits observed in FASD; and (2) DNA methylation patterns in peripheral tissue reflect some changes in brain, which could serve as potential biomarkers for central alterations induced by PAE.

## Materials and Methods

### Prenatal Treatment

Details of the procedures for breeding and handling have been published previously (Bodnar et al., 2016). Briefly, outbred male and nulliparous female Sprague-Dawley rats were obtained from Charles River Laboratories (St. Constant, Quebec, Canada). Following a one-week habituation period, each female (*n* = 39) was pair-housed with a male and vaginal lavage samples were collected daily for estrous cycle staging and to check for the presence of sperm, indicating gestation day (GD) 1. Pregnant dams were singly housed and assigned to one of three prenatal treatment groups: Prenatal alcohol exposure (PAE)—*ad libitum* access to liquid ethanol diet, 36% ethanol-derived calories, 6.37% v/v, *n* = 13; Pair-fed (PF)—liquid-control diet, maltose-dextrin isocalorically substituted for ethanol, in the amount consumed by an PAE partner, g/kg body weight/GD, *n* = 14; or Control (C)—pelleted version of the liquid control diet, *ad libitum, n* = 12. All animals had *ad libitum* access to water. Experimental diets (Weinberg/Keiver Liquid Ethanol Diet #710324, Weinberg/Keiver Liquid Control Diet #710109, and Pelleted Control Diet #102698, Dyets Inc., Bethlehem, PA) were fed from gestation days 1–21, and then replaced with laboratory chow. Litters were weighed and culled at birth to 6 males and 6 females, when possible. Pregnancy outcomes and body weights can be found in Supplementary Table 1. Blood alcohol levels were measured as previously reported and ranged from 80 to 150 mg/dl in PAE females (Hellemans et al., 2010b; Uban et al., 2010; Bodnar et al., 2016).

### Sample Collection

**Figure 1:**
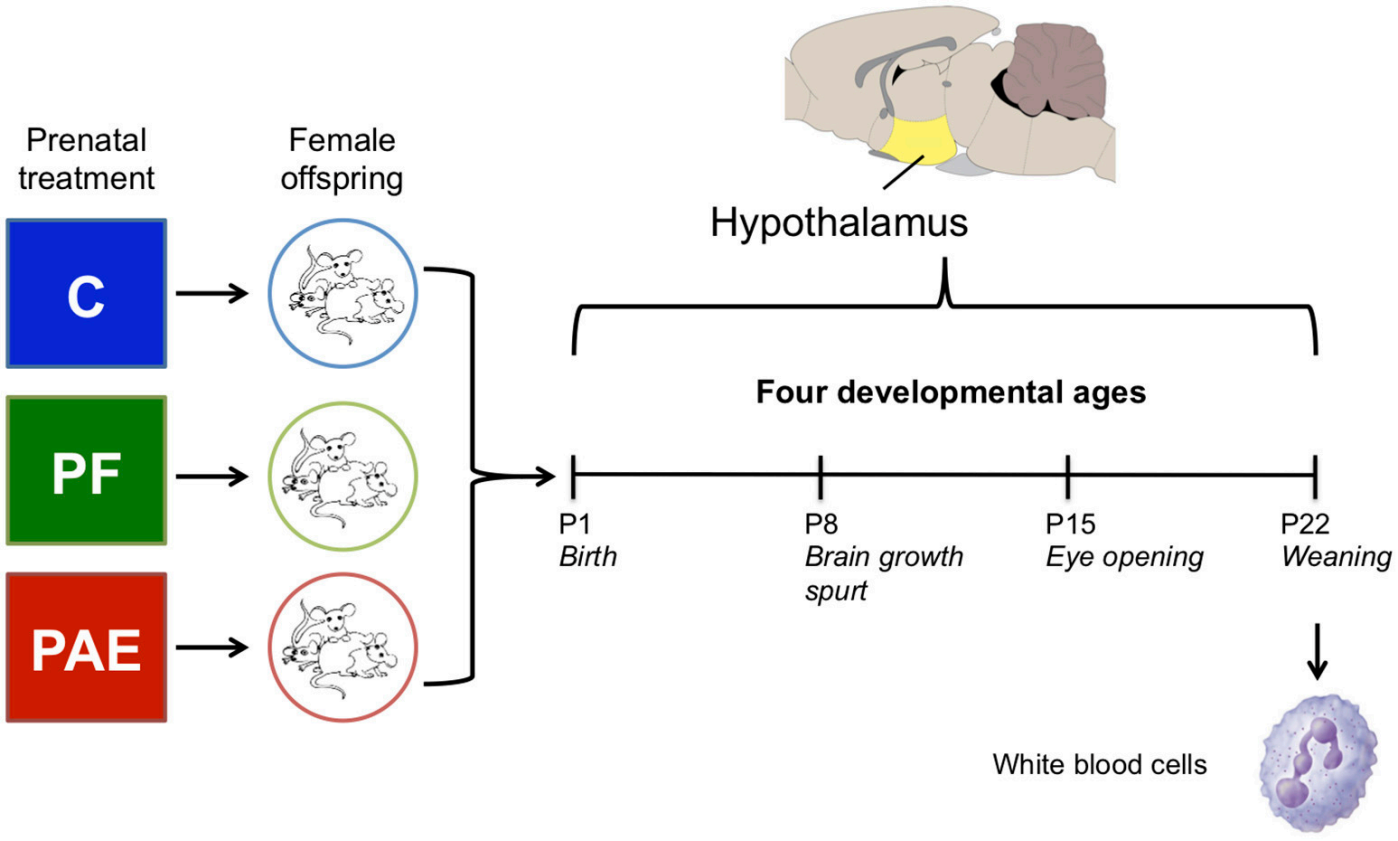
Overview of the experimental design. We collected the hypothalamus of female offspring from one of three prenatal treatment groups on postnatal days (P) 1, 8, 15, and 22. In parallel, white blood cells were collected on P22 from the same animals as the hypothalamus samples. Each group/age/tissue was composed of four samples for DNA methylation analysis by methylated DNA immunoprecipitated and next-generation sequencing (meDIP-seq).

We focused our investigation of PAE-induced epigenetic alterations on female animals, as sexually dimorphic effects of PAE are widely reported (Lee and Rivier, 1996; Weinberg et al., 2008) and females are generally underrepresented in molecular and genome-wide studies of FASD (Lussier et al., 2017). On postnatal day (P) 1, 8, 15, and 22 female offspring (max 1/litter) were decapitated, trunk blood collected (at P22 only), and brains removed and weighed; the hypothalamus was then quickly dissected and frozen on dry ice in RNA*later* (*n* = 7–11/age/group; Figure 1; Qiagen, Hilden, Germany). WBCs were isolated using *Ficoll-Paque* (GE Healthcare, Uppsala, Sweden), which isolates peripheral blood mononuclear cells (PBMCs). All tissue collected was left at 4°C for 24 h to allow for complete permeation by RNA*later* and then frozen at −80°C until DNA extraction, as per manufacturer instructions. WBCs were stored in RNA*later* at −80°C until DNA extraction (Qiagen, Hilden, Germany). Due to the large number of animals associated with the experimental design of this study, animals were collected across four different cohorts (experimental breedings), spanning January 2012–December 2013.

### Blood Composition Analysis

Analysis of blood composition was done on samples from a separate cohort of animals, which were bred at a later time but under the same conditions as the main cohort. Briefly, on P22, trunk blood was collected from female offspring (C: *n* = 6; PF: *n* = 5; PAE: *n* = 5), and analyzed using the Advia120 hematology system, which assesses complete blood counts and differential WBC counts (CBC/Diff function). The reported values include counts for neutrophils, lymphocytes, monocytes, eosinophils, basophils, and large unclassified cells (Supplementary Table 2).

### DNA Extraction

Total RNA and DNA were simultaneously extracted from the hypothalamus and white blood cells (*n* = 4/group/age/tissue; Qiagen AllPrep DNA/RNA Mini kit, Hilden, Germany). Frozen tissue was thawed on ice, quickly weight, and placed in lysis buffer for 5 min. Homogenization was performed by 5 strokes of an 18 G needle, 10 strokes of a 20 G needle, and 10 strokes of a 23 G needle. The resulting homogenate was centrifuged at 21,000 g for 3 min and the supernatant was collected for DNA and RNA extraction. White blood cells were thawed on ice and then centrifuged at 10,000 g for 10 min. RNA*later* was carefully removed without disturbing the cell pellet and cells were resuspended in lysis buffer. The cells were then frozen at −80°C to disrupt cell membranes and thawed on ice. The resulting homogenate was used for DNA and RNA extraction. DNA concentration was assessed using Qubit Fluorometric Quantitation (Life Technologies, Carlsbad, USA). Full developmental data on the animals can be found in Supplementary Table 3.

### Methylated DNA Immunoprecipitation and Next-Generation Sequencing

Our methylated DNA immunoprecipitation followed by next-generation sequencing (meDIP-seq) procedures were adapted from a previously published protocol and are outlined in detail below (Taiwo et al., 2012). Importantly, meDIP-seq provides qualitative measures of methylated CpG levels genome-wide and, unlike bisulfite dependent assays, is specific for methyl-cytosine. As previously noted, meDIP-seq coverage is highly reproducible with reduced coverage in regions of sparse CpG density, showing >99% concordance with bisulfite sequencing methods (Harris et al., 2010). However, our experimental design is based on treatment vs. controls and thus only queries genomic regions that are addressable by meDIP-seq reads. As such, we cannot eliminate the possibility that additional DMRs are present within genomic regions with sparse CpG coverage.

#### Sequencing Library Preparation

For each sample, 500 ng of DNA were diluted in a total volume of 60 μL of EB buffer (Qiagen, Hilden, Germany). DNA was then transferred to a 96-well plate and sheared for 1 h using the Covaris Focused-ultrasonicator. DNA was purified using Ampure XP in 20% polyethylene glycol (PEG) beads to obtain fragments sized from 200 to 500 basepairs (Beckman-Coulter, Brea, USA). Library preparation was performed on the Bravo Automated Liquid Handling Platform (Agilent, Santa Clara, USA) using the TruSeq DNA PCR-Free Sample Preparation Kit (Illumina, San Diego, USA). Following end-repair and A-tailing, adapters were ligated overnight at room temperature. PCR-free library preparation allowed for the conservation of methylated cytosines for subsequent methylated DNA immunoprecipitation. Finally, DNA was resuspended in 35 μL of EB buffer (Qiagen, Hilden, Germany). DNA was quality controlled using Qubit Fluorometric Quantitation and the DNA 1000 Bioanalyzer 2100 kit (Agilent, Santa Clara, USA) to verify DNA concentration and fragment size (250–550 bp).

#### Methylated DNA Immunoprecipitation

For each sample, 400 ng of the sequencing library DNA were diluted in a total volume of 50 μL of IP Buffer (10 mM sodium phosphate buffer, pH 7.0, 140 mM NaCl, 0.05% triton). DNA was denatured by incubation at 95°C for 10 min, followed by the addition of 48 μL ice-cold IP buffer and incubation on ice for 10 min. Two microliters of anti-5-methylcytosine antibody (Eurogenetec, Liège, Belgium), diluted to 1/50 in IP buffer (1 μL of antibody per 1 μg of DNA ratio), were added to each sample. Immunoprecipitation reactions were incubated for 16 h at 4°C with overhead rotation. Following two 5 min washes with 150 μL of 0.1% BSA/PBS, 50 μL of Dynabeads Protein G were incubated with 5 μL of secondary antibody (rabbit anti-mouse IgG; Jackson Immunoresearch, West Grove, USA) in 45 μL ice-cold IP buffer for 15 min at room temperature with overhead rotation. Beads were washed twice with IP buffer to remove unbound secondary antibody and resuspended in 50 μL IP buffer. The antibody-bound beads were added to the immunoprecipitation reactions and incubated for 2 h at 4°C with overhead rotation. Beads were then washed 6 times with 150 μL of ice-cold IP buffer and resuspended in 98.97 μL of Proteinase K digestion buffer (TE with 0.5% SDS). Following the addition of 1.25 μL Proteinase K (20 mg/mL; Qiagen, Hilden, Germany), samples were incubated in a thermomixer for 2 h at 55°C with a rotation speed of 1,250 rpm. The reaction was then allowed to cool at room temperature for 15 min. Supernatant was collected and bead cleanup was performed using equal volume SeraMag beads with 30% PEG. DNA was resuspended in 35 μL of EB buffer (Qiagen, Hilden, Germany).

#### Sample Amplification and Indexing

To reduce PCR amplification bias, two separate reactions of the same meDIP sample were run in parallel using the following PCR amplification cycle conditions. The reaction mixes were as follows: 15 μL DNA, 27 μL H_2_O, 12 μL 5X HF buffer, 1.5 μL DMSO, 1.0 μL paired-end primer (Illumina), 0.5 μL Phusion High-Fidelity DNA polymerase (New England Biolabs), 2 μL indexing primer (Illumina—specific to each sample). The amplification cycle was as follows: 98°C for 1 min, 12X (98°C for 15 s, 65°C for 30 s, 72°C for 30 s), 72°C for 5 min. Reactions from the same sample were pooled and bead cleanup was performed using SeraMag beads in 20% PEG (102 μL of beads per 120 μL of reaction). DNA was resuspended in a final volume of 35 μL of EB buffer.

#### Next-Generation Sequencing and Quality Control

Indexed meDIP libraries were combined in 3 pools of 20 samples each, distributing samples evenly by tissue, age, and prenatal treatment across all three sets. Next-generation sequencing was performed on the pools by the Genome Sciences Centre in Vancouver, BC, Canada. Each sample pool was run on two HiSeq lanes, which produced approximately 600,000,000 paired-end reads of 125 bases per lane. Fastq files were aligned to the most current rat genome (Rn6, July 2014) using the Burrows-Wheeler Transform (BWA) tool to obtain.bam files (Li and Durbin, 2009). Bam files were filtered using *samtools* to remove duplicate reads, unpaired reads, and reads with a minimum quality score below 10. Following alignment and filtering, each the two runs for each sample were merged using *samtools* to obtain a single bam file for each sample (Li et al., 2009). Supplementary Table 11 shows sequencing related information: sample pool, sample index, number of raw reads, number of filtered reads, and total number of reads/sample.

### Bioinformatic Analyses

#### Peakset Generation

Model-based analysis of ChIP-seq (MACS2; version 2.1.0.20140616) was used to identify enriched regions of DNA methylation across the genome (Zhang et al., 2008). The peak calling to identify peak regions (DNA methylation windows) was performed using the “callpeaks” function on paired end bam files with no control input and the following options: –f BAMPE–m 5 50–bw 300–g 2.9e9–q 0.05. Each sample was modeled individually, generating 60 total peaksets. These were imported into R using the *DiffBind* package (Stark and Brown, 2011). As all samples had slightly different predicted peaks, peaksets were combined into common regions using the *dba.count* function in *DiffBind*, which removed peaks found in < 3 samples across the entire dataset and provided the total number of reads within each peak/sample. This created a final dataset of 469,162 peaks and 48 samples from the developmental profile of the hypothalamus, and a final dataset of 350,960 peaks and 24 samples in the P22 hypothalamus and WBC (tissue-concordant) peakset.

#### Data Preprocessing and Normalization of the Developmental Dataset

First, the total reads within each peak were adjusted to reads/kilobase by dividing the number of reads within each region by their length. In turn, these were converted to reads per kilobase per million (RPKM) by dividing the reads/kilobase by the total number of reads found in the predicted peaks to account for differences in sequencing depth between samples. The samples in the developmental dataset were highly correlated (*r* > 0.95 for all samples), with samples clustering most closely with animals of the same age (Supplementary Figure 1). No outliers were detected in this first pass analysis.

Principal component analysis of the normalized RPKM data revealed significant levels of variation associated with batch effects. Notably, meDIP and DNA extraction rounds were associated with a large proportion of variation within the dataset. However, both these factors were highly confounded with age, as samples for each age were extracted as a batch and all P22 samples were immunoprecipitated in a separate batch (Supplementary Figure 2). To account for these effects, ComBat correction, from the *SVA* package, was performed on the RPKM data from the hypothalamic samples to correct the effects of meDIP round and DNA extraction round in the dataset (Leek et al., 2012). Age was also slightly confounded with the breeding from which animals were collected, as not all ages were sampled from each of the different cohorts. Interestingly, some partial effects of breeding remained in the dataset following ComBat correction, suggesting that this covariate was not fully confounded with age. Furthermore, prenatal treatment accounted for a larger proportion of variance within the dataset following ComBat correction, suggesting that the removal of batch effects might allow for the identification of more subtle effects of PAE. The corrected and normalized RPKM values obtained from ComBat were used for plotting purposes, but were converted back to reads/kilobase for downstream statistical analyses.

#### Data Preprocessing and Normalization of the Tissue-Concordant Dataset

The tissue-concordant dataset was preprocessed and normalized as described above. Samples were highly correlated within tissue (*r* > 0.96), the main driver of DNA methylation patterns, and well correlated within the same animals (*r* > 0.92). However, one PF WBC sample clustered with the hypothalamus samples, suggesting that it may have been mislabeled during processing. As such, this sample was removed from the dataset, resulting in a dataset of 23 samples (Supplementary Figure 3). Principal component analysis of the normalized tissue-concordant RPKM data revealed significant levels of variation associated with DNA extraction round batch effects (Supplementary Figure 4). Tissue type was the covariate most strongly associated with variance in the dataset, although it was slightly confounded with extraction round. While ComBat correction was used to account for the effects of DNA extraction round in the tissue-concordant dataset, this approach limited our ability to identify tissue-specific differences, as it removed the majority of tissue-associated variance from the dataset. Again, prenatal treatment was associated with a larger proportion of variance within the dataset following ComBat correction. Interestingly, breeding once again remained a major contributor to variability within the dataset, suggesting that differences between cohorts may have an important influence on epigenetic patterns.

#### Removing Cell-Type Specific DMRs

Using previously characterized transcriptomic profiles from mouse neurons, oligodendrocytes, and astrocytes, we identified DNA methylation peaks within genes that are specifically expressed in each different cellular subtype (1.5-fold expression difference compared to other cell types; Cahoy et al., 2008). Given the relationship between gene expression and epigenetic patterns, it is possible that alterations to the DNA methylation levels of these genes could reflect changes in the cell-type proportions within this dataset. However, the majority of the peaks in the dataset were located within intergenic regions, with no annotated associations with these genes, reducing our ability to capture cell-type related differences. As such, only regions directly located within neuron-, oligodendrocyte-, or astrocyte-specific genes were removed from further analyses to reduce the potential confounding factor of cell type, resulting in a dataset of 451,112 peaks for downstream analyses of the hypothalamus.

#### DMR Identification

Linear modeling was performed using *edgeR*, which is typically used to analyze RNA-seq count data and includes a factor to account for the number of reads in each sample (Robinson et al., 2010a; Nikolayeva and Robinson, 2014). This method was used to identify differentially methylated regions (DMRs) that were consistently different between PAE animals and both control groups across the different ages and tissues. For both analyses, the model accounted for the effects of collection during different breedings, and *p*-values were corrected for multiple-testing using the Benjamini-Hochberg method. Statistically significant DMRS at a false discovery rate (FDR) < 0.05 were obtained for the following contrasts: PAE vs. C, PAE vs. PF, and C vs. PF. The final PAE-specific DMRs were statistically significant in both PAEvC and PAEvPF, and were not found in the PFvC contrasts.

#### Genomic Enrichment

Custom annotations were built for each peakset using the UCSC genome browser gene annotations. Briefly, genomic coordinates of all CpG islands, exons, introns, promoters (TSS-200 bp and TSS-1500 bp), 3′ untranslated regions (UTR), 5′ UTRs for the rn6 genome were obtained as bed files from the table browser. In parallel, MeDIP-seq peaks were converted to the bed file format and the overlap of genomic features with MeDIP-seq peaks was computed iteratively using the *intersectBed* function from *bedtools*, retaining only the peaks that contained the assessed genomic feature (Quinlan and Hall, 2010). The overlaps were concatenated into a single annotation set in R, where individual peaks contained information for each potential genomic feature. Of note, regions spanning both introns and exons were deemed intron/exons boundaries. *P*-values for genomic feature enrichment analyses were calculated by computing background levels of genomic features on 1,000 random subsets of DMRs, using the same number of PAE-specific DMRs.

#### Transcription Factor Binding Site Analysis

Enrichment of different transcription factors binding sites (TFBS) in PAE-specific DMRs was assessed using the *motifEnrichment* function of the *PWMEnrich* package (Stojnic and Diez, 2013). DMR DNA sequences were obtained from the UCSC genome browser (Rn6 genome). As no binding motifs were available for the *Rattus norvegicus* genome, motifs from the *Mus musculus* genome were obtained from the *PWMEnrich.Mmusculus.background*. Motifs were summarized using the *groupReport* function. *P*-values were calculated by performing enrichment analysis on 1,000 random subsets of DMRs, using the same number of PAE-specific DMRs for each analysis to assess background levels of each TFBS in the different peaksets.

#### Gene Ontology Analysis

The gene-score resampling (GSR) tool of ErmineJ (version 3.0.2) was used to identify gene function enrichment in the differentially methylated genes including the Gene Ontology (GO) annotations molecular function, biological process, and cellular component (Lee et al., 2005; Gillis et al., 2010). The ermineJ GSR tool was set with the following parameters: max gene set size = 2,000; min gene set size = 2; iterations = 10,000. Once again, statistically significant associations (*p* < 0.05 and multifunctionality score < 0.05) were obtained for the following contrasts: PAE vs. C, PAE vs. PF, and C vs. PF. The final PAE-specific GO terms were statistically significant in both PAEvC and PAEvPF, and were not found in the PFvC contrasts. Importantly, ermineJ accounts for the multiple functions assigned to many genes, generating a multifunctionality *p*-value to reduce the bias of gene ontology analyses toward pathways with numerous common genes (Gillis and Pavlidis, 2011; Ballouz et al., 2017).

### Bisulfite Pyrosequencing

DNA from the same samples as above were subjected to bisulfite conversion using the Zymo EZ DNA Methylation Kit (Zymo Research, Irvine, California), which converts DNA methylation information into sequence base differences by deaminating unmethylated cytosines to uracil while leaving methylated cytosines unchanged. Bisulfite pyrosequencing assays were designed with PyroMark Assay Design 2.0 (Qiagen, Hilden, Germany; Supplementary Table 4). The regions of interest were amplified by PCR using the HotstarTaq DNA polymerase kit (Qiagen, Hilden, Germany) as follows: 15 min at 95°C, 45 cycles of 95°C for 30 s, 58°C for 30 s, and 72°C for 30 s, and a 5 min 72°C final extension step. For pyrosequencing, single-stranded DNA was prepared from the PCR product with the Pyromark™ Vacuum Prep Workstation (Qiagen, Hilden, Germany) and the sequencing was performed using sequencing primers on a Pyromark™ Q96 MD pyrosequencer (Qiagen, Hilden, Germany). The quantitative levels of methylation for each CpG dinucleotide were calculated with Pyro Q-CpG software (Qiagen, Hilden, Germany). Of note, only PAE and C animals were assessed by bisulfite pyrosequencing. We selected several DMRs for verification by bisulfite pyrosequencing based on their potential role in PAE-induced deficits, mainly focusing on their associated gene.

## Results

### The Developmental Profile of the Rat Hypothalamus

Our initial analysis of this dataset aimed to identify persistent alterations to DNA methylation patterns in the female rat hypothalamus across early development (P1, 8, 15, and 22) using methylated DNA immunoprecipitation (meDIP-seq). These ages were selected as they represent key developmental time points, including birth (P1), the brain growth spurt (P8), eye opening (P15), and weaning (P22; Dobbing and Sands, 1979; McCormick and Mathews, 2010).

#### PAE Resulted in Persistent Alterations to DNA Methylation Patterns in the Hypothalamus

As cell type proportions are a major driver of DNA methylation patterns, we first removed peaks that were located within genes specifically expressed in neurons, astrocytes, or oligodendrocytes, resulting in a dataset of 48 samples and 451,112 peaks. We assessed the cell-type associated peaks independently by linear modeling (18,050 peaks), identifying few differences among prenatal groups (Supplementary Figure 5). Although this filter was limited by the use of genic and promoter regions, as well as gene expression patterns from isolated neurons, glia, and oligodendrocytes, this partial analysis suggested that few differences in the proportions of the three major brain cell types were present in the dataset. In addition to cell type differences, highly-methylated regions associated with age could have also introduced additional variation into our data. As such, we included age as a covariate in our statistical models, which may partially account for these age-induced effects through statistical means.

**Figure 2:**
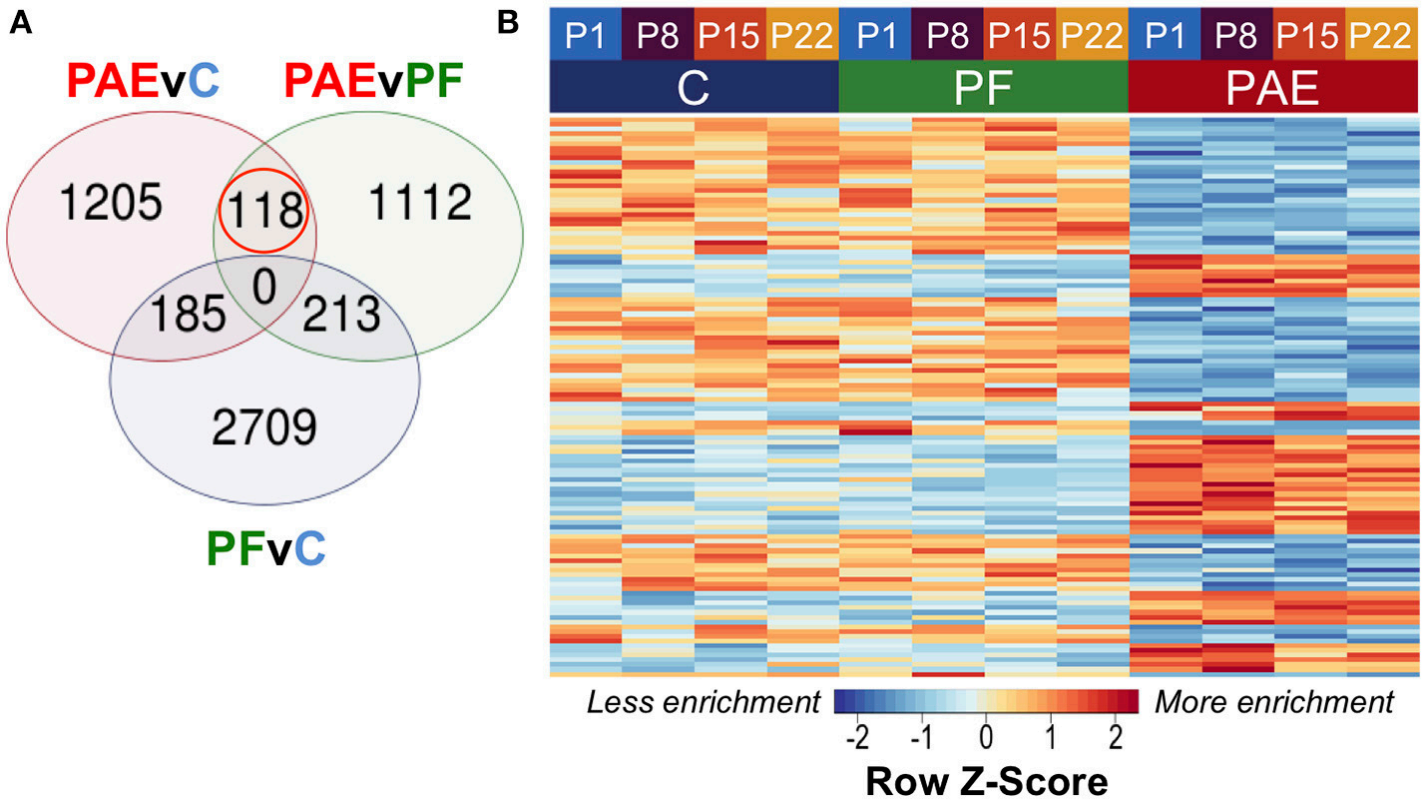
PAE-specific DMRs across pre-weaning development of the hypothalamus. **(A)** Contrast analysis revealed 118 PAE-specific differentially methylated regions (DMR), which were significantly different in PAE vs. C animals and PAE vs. PF animals, but not significantly different between PF vs. C. **(B)** The DMRs showed consistent difference between PAE animals and controls across ages. Each row represents a different DMR, while each column shows the mean for all animals within that group/age (*n* = 4). Reads per kilobase per million (RPKM) data were scaled and centered to produce a Z-score for each DMR, where those in blue showed less DNA methylation enrichment and those in red showed more enrichment.

To assess persistent alterations to DNA methylation patterns caused by PAE, we performed linear modeling on the hypothalamic samples across all ages with a model that also accounted for differences across breeding cohorts. Using contrast analyses, we successfully identified 118 PAE-specific DMRs at an FDR < 0.05 that persisted across all four developmental ages and showed consistently different DNA methylation levels between PAE animals and controls (Figure 2; Supplementary Table 5). Of these, 47 were up-methylated and 75 were down-methylated in PAE animals vs. control groups, and their sizes ranged from 316 to 1027 bp (median = 494.5 bp).

**Figure 3:**
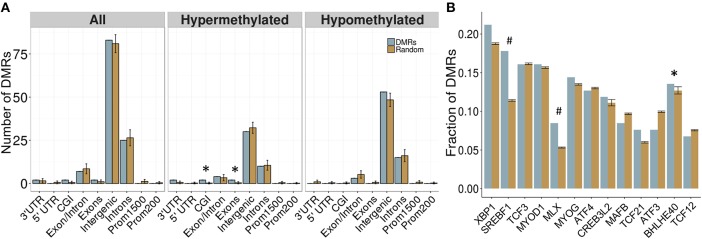
Enrichment patterns of the developmental DMRs. **(A)** Genomic feature enrichment profile of all, up-methylated, and down-methylated DMRs. The probe counts for each feature (blue) were compared to the results from permutation analyses of 118 random regions (orange), which were used to compute the *p*-value. The majority of DMRs were located in intergenic regions or introns. Up-methylated regions in PAE animals contained more CpG islands (CGI) and exons than expected by chance (*p* < 0.05). **(B)** Overrepresentation analysis of transcription factor binding sites in the DMRs. Only BHLHE40 showed higher enrichment in the PAE-specific DMRs (blue) than by random chance (orange; *p* < 0.05), although SREBF1 and MLX trended toward significance (*p* < 0.1). *p < 0.05, #*p* < 0.1.

Overall, 34 DMRs were located in genes, particularly within those involved in dopamine signaling (*Drd4*), the immune response (*Ifih1, Ccrl2, Il20ra*), and blood-brain barrier function (*Plvap*). Of note, two overlapping genes, *Golga4* and *Ctdspl*, contained two separate DMRs, and were the only genes with multiple DMRs. Although the entire DMR set did not show any significant differences in genomic location enrichment compared to the background of the dataset, the up-methylated DMRs displayed significantly more enrichment in CGI and exons compared to random chance (*p* < 0.05; Figure 3). Furthermore, the majority of DMRs were located in intergenic regions, and while these were not significantly enriched compared to the entire dataset, these results suggested that intergenic regions may be more responsive to the influence of PAE on the epigenome, and may contain important regulatory regions that are not yet annotated in the rat genome.

Of note, meDIP-seq provides relative levels of DNA methylation based on enrichment scores, and thus, the magnitude of change (i.e., % methylation) was not assessed using this method. Nevertheless, 38 of the DMRs showed at least 1.5-fold change in DNA methylation levels in PAE animals vs. controls, including those in *Drd4, Plvap*, and *Cntnap5c* (Supplementary Table 6), suggesting that PAE could induce robust alterations to DNA methylation patterns in the hypothalamus.

#### PAE-Specific DMRs Contained a Greater Proportion of Computationally-Predicted Bhlhe40 and Srebf1 Transcription Factor Binding Sites

To follow up on the large proportion of intergenic regions in the PAE-specific DMRs, we assessed the enrichment of transcription factor binding sites (TFBS) within these regions using binding motifs from the mouse genomes. Although the overlap between the rat and mouse genomes is not perfect (~70%), the rodent family shares many genomic characteristics and this analysis provided an important first pass analysis of potential regulatory factors within these regions. Following multiple-test correction (FDR < 0.05), few TFBS were enriched within these regions compared to background levels. However, the BHLHE40 binding motif was significantly enriched within the PAE-specific DMRs (*p* < 0.05), while the SREBF1 and MLX motifs trended toward significance (*p* < 0.10; Figure 3B). These results suggest that certain transcription factors may play a role in the long-term reprogramming of hypothalamic functions by PAE and may act in concert with other factors to sculpt the epigenome and downstream phenotypes.

#### Genes in PAE-Specific DMRs Were Enriched for Biological Processes Associated With Hypothalamic Functions

We performed GO analysis to ascertain the broad functional impact of PAE-induced changes in DNA methylation patterns of the hypothalamus across early development. This analysis revealed 20 PAE-specific biological processes (both *p*-values and multifunctionality *p*-value < 0.05 in PAEvC and PAEvPF, >0.05 in PFvC; Table 1). Although these findings failed to reach significance after multiple-test correction, they were potentially reflective of the broader effects of PAE on the epigenome. Of note, the top GO terms were associated with steroid receptor signaling (GO:0042921, GO:0030518, GO:0031958, GO:0030520), a key function of the hypothalamus. Several processes associated with epigenetic regulation (GO:0016577, GO:0006482, GO:0070932) were also enriched in the PAE-specific DMRs, as were processes involved in immune function (GO:0030885, GO:0030886, GO:0002314), and cellular metabolism (GO:0050812).

**Table 1:** Biological processes enriched in the developmental profile DMRs.

**Name**	**ID**	**Number of genes**	**Multi-functionality**	***P*****-value**			**Multifunctionality** ***p*****-value**		
				**PAEvC**	**PAEvPF**	**PFvC**	**PAEvC**	**PAEvPF**	**PFvC**
Glucocorticoid receptor signaling pathway	0042921	4	0.475	0.00117	0.00432	0.06853	0.0011	0.00456	0.07096
Intracellular steroid hormone receptor signaling pathway	0030518	27	0.681	0.00146	0.00865	0.09115	0.00148	0.00765	0.09009
Corticosteroid receptor signaling pathway	0031958	5	0.442	0.0025	0.01919	0.10193	0.00269	0.01955	0.1019
Regulation of myeloid dendritic cell activation	0030885	2	0.129	0.00816	0.0198	0.14194	0.00843	0.01869	0.14077
Negative regulation of myeloid dendritic cell activation	0030886	2	0.129	0.00816	0.02063	0.1637	0.00843	0.01978	0.163
Histone demethylation	0016577	13	0.397	0.01224	0.02727	0.18051	0.01204	0.02756	0.17928
Protein demethylation	0006482	15	0.365	0.01636	0.0284	0.18496	0.01597	0.02785	0.18571
Protein dealkylation	0008214	15	0.365	0.01636	0.02926	0.2578	0.01597	0.02805	0.26243
Calcium ion export	1901660	3	0.345	0.0166	0.02927	0.32371	0.01739	0.02926	0.32667
Protein sumoylation	0016925	11	0.328	0.01845	0.03449	0.33119	0.01688	0.03389	0.33539
Regulation of protein targeting to membrane	0090313	11	0.631	0.01845	0.03449	0.42205	0.01688	0.03389	0.42409
Intracellular estrogen receptor signaling pathway	0030520	6	0.523	0.01891	0.0354	0.42205	0.01913	0.0354	0.42409
Histone H3 deacetylation	0070932	8	0.419	0.02819	0.04091	0.56227	0.03028	0.04161	0.56796
Relaxation of smooth muscle	0044557	6	0.679	0.03185	0.04117	0.56279	0.03219	0.04121	0.56348
Midbrain-hindbrain boundary development	0030917	3	0.267	0.03285	0.04178	0.72697	0.03382	0.04319	0.72413
GDP-mannose metabolic process	0019673	5	0.252	0.03451	0.04275	0.7401	0.03368	0.04445	0.73884
Protein deacetylation	0006476	20	0.664	0.04114	0.04456	0.76723	0.04009	0.04284	0.76878
Regulation of acyl-CoA biosynthetic process	0050812	4	0.358	0.04459	0.04469	0.89405	0.04633	0.04556	0.89682
Germinal center B cell differentiation	0002314	2	0.073	0.0467	0.04469	0.89405	0.04618	0.04556	0.89682
Negative regulation of nuclear division	0051784	24	0.774	0.04736	0.04683	0.97779	0.04927	0.04737	0.97771

#### The Ddr4 DMR Was Verified by Bisulfite Pyrosequencing

Given that meDIP-seq provides a relative signal of DNA methylation levels, we verified the PAE-specific DMRs using bisulfite pyrosequencing, a highly quantitative measure of DNA methylation, to test whether meDIP-seq could accurately detect alterations in DNA methylation patterns. Importantly, this technique also detects DNA hydroxymethylation, but cannot differentiate between the different cytosine modifications, while meDIP-seq is specific to DNA methylation due to the nature of the antibody used. We assessed four different DMRs, based on their potential role in the etiology of PAE-induced deficits. Of note, only a portion of CpGs within each DMR were assessed by bisulfite pyrosequencing due to limitations in read length, and as such, additional CpGs within the DMR may partially drive some of the differential DNA methylation enrichment identified by meDIP-seq.

**Figure 4:**
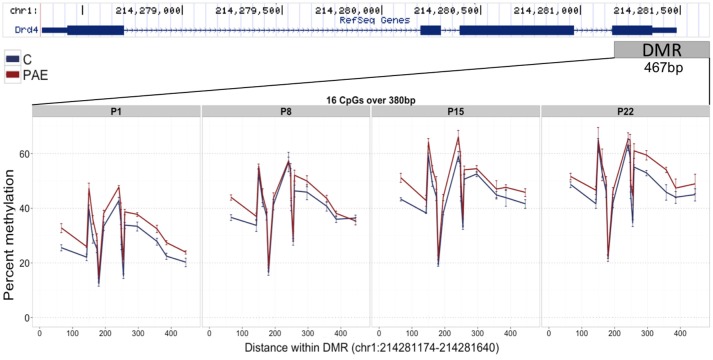
Bisulfite pyrosequencing verification of the *Drd4* DMR. 16 CpGs (#7–22) spanning 380 base pairs (bp) of the DMR located in the 3′ UTR of *Drd4* were verified by pyrosequencing in the same animals as the meDIP-seq analysis. All CpGs on P1 displayed >5% change in DNA methylation levels between PAE (red) and controls (blue). Of these, several were consistently different across all ages and a number persisted until P22. The total levels of DNA methylation in the DMR also increased with age across all groups.

We first assayed 16 CpGs within the 3′ UTR of the *Drd4* DMR (chr1:214,281,174-214,281,640) in the same samples as the meDIP-seq analysis (Figure 4; Supplementary Table 7). DNA methylation at all CpGs assayed was significantly associated with PAE when correcting for age (*p* < 0.05). Moreover, this analysis detected a >5% change in DNA methylation across the entire DMR on P1 and P22 in PAE compared to C animals (*p* < 0.05). A 5% DNA methylation change is often used as an arbitrary quantitative cutoff for potential biological significance in epigenome-wide association studies, although we note that many studies report lower effect sizes (Mill and Heijmans, 2013; Breton et al., 2017). At older ages, several of the CpGs remained significantly different between PAE and controls, with several remaining present on P22. Overall, bisulfite pyrosequencing showed the same direction of change as the meDIP-seq analysis in this DMR and generally displayed good concordance between the two technologies (Table 2).

**Table 2:** Summary of the pyrosequencing results for the *Drd4* DMR.

**Age**	**DMR DNAm**		**DMR DNAm change**				**DMR *p*-value**	**Individual CpGs (from #7–22 of the DMR)**		
	**PAE**	**C**	**Mean**	**95% CI (lower)**	**95% CI (upper)**	**SD**		***p* < 0.05**	**change >0.05**	***p* < 0.05 and change >0.05**
All ages	44.310	40.211	3.925	1.549	6.301	1.212	2.59E-03	CpG 7–22	CpG 7, 9, 10, 17, 18	CpG 7, 9, 10, 17, 18
P1	32.868	27.748	5.120	2.371	7.869	1.402	6.49E-03	CpG 7–21	CpG 7, 9, 10, 15–17	CpG 7, 9, 10, 15–17
P8	41.393	41.666	−0.273	−5.214	4.667	2.521	9.16E-01	CpG 7	CpG 7	CpG 7
P15	49.393	44.790	4.603	2.120	7.085	1.267	8.36E-03	CpG 7–10, 12, 13, 15–17	CpG 7, 10, 13, 16	CpG 7, 10, 13, 16
P22	53.587	47.555	6.032	1.452	10.613	2.337	3.25E-02	CpG 8, 13, 16–20	CpG 8–11, 13, 15, 17–22	CpG 8, 13, 17–20

We also used this method to verify three additional DMRs, located within *Ifih1* (chr3: 48,561,559-48,561,925), *Mycbp* (chr5:141,565,784-141,566,172), and *Plvap* (chr16: 19,912,813-19,913,185) (Supplementary Figure 6; Supplementary Table 7). These showed less consistent changes in DNA methylation between the two methods, as some ages appeared to drive DNA methylation patterns more than others and some CpGs showed opposite direction of change between meDIP-seq and pyrosequencing. For instance, the *Ifih1* locus only showed significant differential DNA methylation at P15 only (*p* = 0.044; change = 1.21%), while the *Mycbp* and *Plvap* loci displayed a subset of significant CpGs at various ages. Although the small differences identified among groups suggest that meDIP may be sensitive enough to detect small changes in DNA methylation levels, the direction of change was not always consistent with the meDIP-seq results. These findings also raised the possibility that DNA hydromethylation differences may also be in play within these loci, since this DNA modification shows high prevalence in the brain and that could potentially explain the discrepancies between meDIP-seq and pyrosequencing (Lister et al., 2013).

### Tissue-Concordant Alterations to DNA Methylation Patterns

In parallel to the analysis of DNA methylation in the hypothalamus during early development, we used meDIP-seq to assay DNA methylation in the hypothalamus and WBC of the same P22 females. This analysis aimed to identify tissue-concordant alterations present in both the central nervous system (CNS) and peripheral tissue in response to PAE.

#### White Blood Cell Proportions Were Not Different Across Groups

As noted, cell type proportions are a major driver of epigenetic variability. However, the volume of blood collected from P22 animals was too small to perform both epigenetic and blood composition analyses on the same animals. For this reason, we collected samples from P22 animals from an independent cohort (i.e., bred at a later time, but under the same conditions as those in the present study) to determine whether PAE altered the proportions of WBCs that would be collected using the *Ficoll-Paque* method. Composition analysis of whole blood indicated the proportions of lymphocytes, neutrophils, monocytes, basophils, eosinophils, and large unclassified cells. Linear modeling revealed no significant differences among prenatal treatment groups, suggesting that PAE does not alter the proportion of the major WBC subtypes (Supplementary Figure 7). These findings suggest that WBC proportions likely did not influence differences in DNA methylation patterns between groups in the present dataset.

#### PAE Caused Tissue-Concordant Alterations to DNA Methylation Patterns

**Figure 5:**
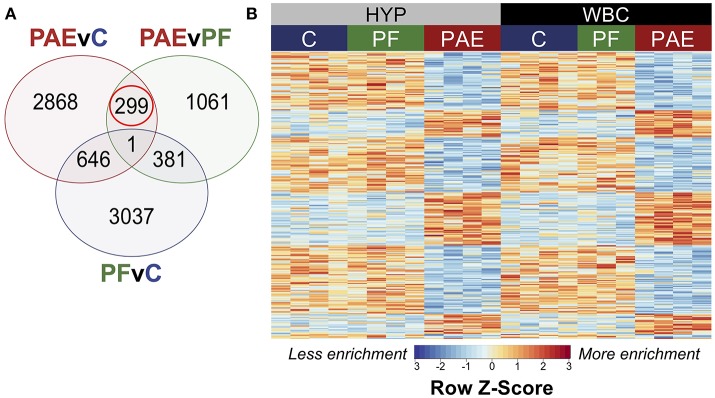
PAE-specific DMRs concordant across the hypothalamus and white blood cells. **(A)** Contrast analysis revealed 299 PAE-specific differentially methylated regions (DMR) between both tissues, which were significantly different in PAE vs. C animals and PAE vs. PF animals, but not significantly different between PF vs. C. **(B)** Heatmap of the DMRs. Each row represents a different DMR, while each column shows the meDIP-seq data for each animal (*n* = 4, except PF WBC: *n* = 3). Reads per kilobase per million (RPKM) data were scaled and centered to produce a Z-score for each DMR, where those in blue showed less DNA methylation enrichment and those in red showed more enrichment. PAE-specific DMRs showed the same direction of change in both tissues, with some graded effects of tissue type.

To identify tissue-concordant alterations to DNA methylation patterns associated with PAE, we performed linear modeling on the tissue-concordant dataset with a model that also accounted for differences across breeding cohorts. This method identified 299 PAE-specific DMRs at an FDR < 0.05 that were present in both tissues and showed the same direction of change in PAE vs. control animals (Figure 5; Supplementary Table 8). In contrast to the developmental profile, these DMRs showed smaller fold changes, with only 7/299 displaying a 2-fold change in PAE animals vs. controls, suggesting that subtle, but potentially important alterations are observed across tissues (Supplementary Table 9).

Of the significant DMRs, 105 were up-methylated and 194 were down-methylated in PAE animals, and their size ranged from 355 to 2,038 bp (median = 574 bp). The majority of DMRs also displayed small tissue-specific effects in the relative enrichment of DNA methylation, although the magnitude of change was similar between PAE and controls across both tissues (Figure 5).

**Figure 6:**
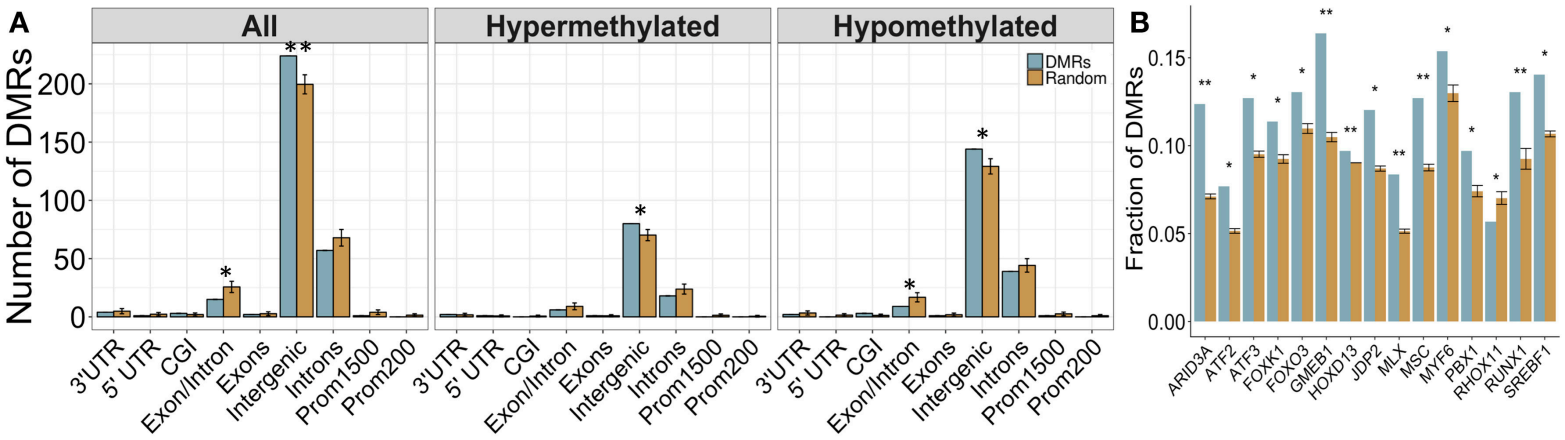
Enrichment patterns of the tissue-concordant DMRs. **(A)** Genomic feature enrichment profile of all, up-methylated, and down-methylated DMRs. The probe counts for each feature (blue) were compared to the results from permutation analyses of 299 random regions (orange), which were used to compute the *p*-value. While the majority of DMRs were located in intergenic regions, they showed a higher proportion than expected by random change (*p* < 0.01). By contrast, exon/intron boundaries were underrepresented in the DMRs, particularly within the regions that were down-methylated in PAE animals. **(B)** Overrepresentation analysis of transcription factor binding sites in the DMRs. Several TFBS showed higher enrichment in the tissue-concordant DMRs (blue) than expected by random chance (orange), with GMEB1 showing the highest enrichment at 16% of all DMRs. *p < 0.05, **p < 0.01.

Again, a majority of DMRs were located in intergenic regions, and were not associated with any gene (Figure 6A). However, the DMRs showed increased enrichment in intergenic regions compared to backgorund levels and less enrichment in intron/exon boundaries, which was driven mainly by the down-methylated regions. These results may reflect the role of DNA methylation in the regulation of splice variants, which could potentially be affected by PAE. Overall, 75 DMRs were located in genes, although the majority of these were once again located in intronic regions. Several DMRs were located in genes involved in immune function (*Fgf9, Il18r1*) and alcohol metabolism (*Adh4*). Of note, one DMR spanned 9 different isoforms of the Utg1a family of genes, which could be related to alternative splicing, while *Caln1* and *Cntnap5c* each contained three separate DMRs.

#### Several Computationally-Predicted TFBS Were Enriched in Cross-Tissue PAE-Specific DMRs

We assessed the enrichment of TFBS within these cross-tissue PAE-specific DMRs to examine potential regulatory regions. Following multiple-test correction (FDR < 0.05), we identified 16 TFBS enriched within these regions compared to background levels (Figure 6B). The most frequent motif belonged to GMEB1, which was found in 16% of all DMRs. Several binding sites for the forkhead box (FOX) family of transcription factors were also enriched in these regions. Of note, the enrichment of MLX and SREBF1 motifs in the cross-tissue DMRs overlapped with the results from the developmental profile.

#### Genes in Cross-Tissue PAE-Specific DMRs Were Enriched for Various Biological Processes

We performed GO analysis to ascertain the broad functional impact of PAE-induced changes in DNA methylation patterns across the hypothalamus and WBC. Through this analysis, we identified 35 PAE-specific biological processes (both *p*-values and multifunctionality *p*-value < 0.05 in PAEvC and PAEvPF, >0.05 in PFvC; Table 3). However, with multiple-test correction, these findings failed to reach significance, suggesting that these may represent more subtle effects on the global epigenome. Of note, the top GO terms were associated with metabolic processes, including aldehyde metabolism (GO:0006081). Several processes were also associated with immune function (GO:0045063, GO:0071351, GO:0032733, GO:0070673, GO:2674), chromatin remodeling (GO:6338, GO:90239), and the stress response (GO:42320).

**Table 3:** Biological processes enriched in the tissue-concordant DMRs.

**Name**	**ID**	**Number of genes**	**Multi-functionality**	***P*****-value**			**Multifunctionality** ***p*****-value**		
				**PAEvC**	**PAEvPF**	**PFvC**	**PAEvC**	**PAEvPF**	**PFvC**
Cellular aldehyde metabolic process	6081	29	0.785	0.00089	0.00081	0.0531	0.00094	0.00093	0.05423
T-helper 1 cell differentiation	45063	5	0.483	0.0026	0.00284	0.0531	0.00261	0.00319	0.05423
Amino-acid betaine metabolic process	6577	10	0.484	0.00275	0.00383	0.05739	0.0028	0.0034	0.058
Carnitine metabolic process	9437	7	0.36	0.00284	0.00414	0.06279	0.00321	0.00391	0.06181
Osteoblast fate commitment	2051	2	0.224	0.0044	0.00465	0.09845	0.00413	0.00434	0.09938
Plasma membrane repair	1778	7	0.109	0.00599	0.0051	0.1162	0.00631	0.00474	0.11789
Negative regulation of circadian sleep/wake cycle, REM sleep	42322	2	0.324	0.00788	0.0051	0.14968	0.00829	0.00474	0.15006
Chromatin remodeling	6338	43	0.753	0.01171	0.00597	0.17051	0.01135	0.00569	0.17029
Negative regulation of axon regeneration	48681	3	0.41	0.01139	0.0092	0.17521	0.01204	0.00896	0.17422
Regulation of natural killer cell cytokine production	2727	2	0.293	0.01217	0.01155	0.17521	0.01348	0.01048	0.17422
Positive regulation of natural killer cell cytokine production	2729	2	0.293	0.01217	0.01082	0.22896	0.01348	0.01057	0.2317
Amino-acid betaine biosynthetic process	6578	5	0.219	0.01428	0.01082	0.25627	0.01367	0.01057	0.25577
Glucose 1-phosphate metabolic process	19255	2	0.0827	0.01405	0.0106	0.29844	0.01546	0.01064	0.30042
Cellular response to interleukin-18	71351	2	0.23	0.01587	0.0114	0.31438	0.01748	0.01094	0.317
Protein K63-linked deubiquitination	70536	12	0.126	0.02072	0.01396	0.33657	0.02115	0.01351	0.33588
Carnitine biosynthetic process	45329	3	0.085	0.02242	0.01864	0.34572	0.02301	0.02005	0.34264
Positive regulation of interleukin-10 production	32733	15	0.81	0.02457	0.02429	0.34572	0.02509	0.02327	0.34264
Response to jasmonic acid	9753	3	0.405	0.02597	0.02429	0.37241	0.02675	0.02327	0.36841
Cellular response to jasmonic acid stimulus	71395	3	0.405	0.02597	0.02776	0.38417	0.02675	0.02755	0.38807
Response to interleukin-18	70673	3	0.404	0.02741	0.03021	0.44431	0.02814	0.02905	0.44259
Cofactor catabolic process	51187	13	0.638	0.0291	0.03294	0.44477	0.02836	0.03162	0.44499
Extracellular polysaccharide biosynthetic process	45226	2	0.12	0.02809	0.03374	0.50515	0.02987	0.03253	0.50571
Extracellular polysaccharide metabolic process	46379	2	0.12	0.02809	0.03382	0.53397	0.02987	0.03259	0.53274
Acetaldehyde metabolic process	6117	2	0.216	0.03048	0.03547	0.53397	0.03224	0.03553	0.53274
Protein K48-linked deubiquitination	71108	12	0.0357	0.0314	0.03784	0.58202	0.03277	0.03703	0.57955
Cellular response to light stimulus	71482	38	0.821	0.03665	0.03755	0.58809	0.03621	0.03751	0.58519
Podosome assembly	71800	3	0.0518	0.03607	0.03755	0.61294	0.03643	0.03751	0.61412
Micturition	60073	5	0.536	0.04093	0.03865	0.65836	0.03964	0.0376	0.65844
Regulation of histone H4 acetylation	90239	5	0.465	0.04093	0.03969	0.66132	0.03964	0.03965	0.66174
Adenylate cyclase-activating G-protein coupled receptor signaling pathway	7189	26	0.73	0.038	0.04176	0.7519	0.03969	0.04102	0.75163
ER to Golgi ceramide transport	35621	2	0.11	0.03821	0.04171	0.76157	0.03982	0.04149	0.75818
Ceramide transport	35627	2	0.109	0.03821	0.04174	0.81677	0.03982	0.04231	0.81613
Glycolipid transport	46836	2	0.0288	0.03821	0.04318	0.84505	0.03982	0.0426	0.84661
Regulation of circadian sleep/wake cycle, REM sleep	42320	4	0.439	0.04575	0.04318	0.86337	0.04542	0.0426	0.86292
Negative regulation of acute inflammatory response	2674	6	0.674	0.04567	0.04881	0.94037	0.04575	0.04964	0.93962

#### Verification of DMRs by Bisulfite Pyrosequencing

We used bisulfite pyrosequencing to compare quantitative levels of DNA methylation between PAE and Control animals in three cross-tissue DMRs. More specifically, we analyzed DNA methylation in the final exon and 3′ UTR of *Adh4* (chr2:243,719,416-243,720,233), the first exon and 5′ UTR of *Ctnnbip1* (chr5:166,485,057-166,485,637), and the first intron of *Ffg9* (chr15:38,377,629-38,378,027; Supplementary Figure 8; Supplementary Table 10). The main differences in DNA methylation levels were identified between tissues, which sometimes showed different directions of change between PAE and controls. In particular, a single CpG within the *Adh4* DMR showed ~5% methylation difference in the hypothalamus of PAE animals (*p* = 0.011; change = 5.94%), but this effect was not present in WBC or both tissues combined and was in the opposite direction of the meDIP-seq results. Another CpG within the *Adh4* locus showed small, but not statistically significant, changes that were consistent between tissues. This pattern was also observed in the *Fgf9* locus, which suggests subtle but potential systemic effects of PAE. Furthermore, DNA methylation in the *Fgf9* DMR was significantly associated with PAE in the hypothalamus (*p* = 0.031; change = 0.94%), but not both tissues combined or WBC alone. By contrast, the *Ctnnbip1* locus showed opposite, but non-significant, effects between tissues (decreased in the hypothalamus; increased in WBC), with only CpG showing significant differential methylation in WBC alone (*p* = 0.036; change = 5.50%) suggesting that other factors may come into play, such as DNA hydroxymethylation or genetic influences. Moreover, as we did not assess quantitative DNA methylation level across the entire DMR due to pyrosequencing limitations, other CpGs may drive the enrichment patterns previously identified by meDIP-seq.

## Discussion

Alcohol exposure *in utero* has been shown to reprogram physiological and neurobiological systems, increasing the risk of adverse developmental outcomes across the lifespan (Zhang et al., 2005; Mattson et al., 2011; Pei et al., 2011). Given the potential role of epigenetic mechanisms in mediating the long-term effects of PAE, the present study aimed to extend previous work on the influence of *in utero* alcohol exposure on the epigenome, using an animal model of PAE to assess genome-wide DNA methylation patterns in the hypothalamus and WBCs during early postnatal development in females, a group largely underrepresented in neurobiological, molecular and genetic studies. We identified 118 differentially methylated regions (DMRs) that were altered in the hypothalamus of PAE vs. control animals across the pre-weaning period. In parallel, we found 299 DMRs displaying concordant DNA methylation alterations between the hypothalamus and WBCs of PAE animals at weaning. Several differentially methylated genes were functionally related to PAE-induced deficits, having roles in the immune response, neurobiological function, and mental health. Additionally, functional enrichment revealed several PAE-specific biological processes, including those related to immune function, the stress response, and epigenetic regulation. In addition, we identified several transcription factor binding sites (TFBS) that were enriched in the DMRs, which may potentially reflect broader programming effects of PAE on the epigenome. Overall, this study is among the first to compare central and peripheral effects of PAE on DNA methylation patterns in addition to characterizing genome-wide changes in females prenatally exposed to alcohol. Our findings suggest that PAE results in widespread alterations to epigenomic programs in both the CNS and peripheral tissues, with a potential impact on both neurobiological and physiological systems. In addition, we demonstrated that peripheral changes in DNA methylation profiles could serve as a potential biomarker of PAE's effects on the CNS. These findings can also provide insight into other neurodevelopmental and mental health disorders, such as autism, ADHD, depression, and many more, as they share numerous outcomes and comorbidities.

Our initial analysis of the DMRs revealed several differentially methylated genes that could be relevant to PAE-induced deficits. In particular, the dopamine receptor D4 (*Drd4*) gene contained a DMR that persisted across the early developmental period. Given its crucial role in dopaminergic function, as well as interactions among dopaminergic, neuroendocrine, and immune systems, alterations to this gene could reflect broader alterations to signaling in the brain. Interestingly, differential DNA methylation patterns of *Drd4* are also present in the buccal epithelial cells of individuals with FASD, suggesting that this may constitute an association of PAE with the epigenome that replicates across organisms (Fransquet et al., 2016; Portales-Casamar et al., 2016; Lussier et al., 2018). Although these were not identified in central tissues, buccal epithelial cells may act as a passable surrogate tissue for brain in human DNA methylation studies, as they originate from the same germ layer (Lowe et al., 2013; Portales-Casamar et al., 2016). Importantly, in addition to this association with FASD, genetic and epigenetic variation in *Drd4* has been linked to attention deficit hyperactivity disorder (ADHD), schizophrenia, bipolar disorder, substance-use disorders, and several other neurobiological disorders (Bau et al., 2001; Chen et al., 2011; Ptácek et al., 2011; Docherty et al., 2012; Kordi-Tamandani et al., 2013; Zhang et al., 2013; Cheng et al., 2014; Faraone et al., 2014; Dadds et al., 2016; Ji et al., 2016). *Golga4* also contained two PAE-specific DMRs across hypothalamic development, and is overexpressed in the prefrontal cortex of individuals with bipolar disorder (Iwamoto et al., 2004). As a member of the Golgi secretory pathway, it could also potentially influence the secretion of neuropeptides by cells of the hypothalamus, possibly playing a role in altered function or responsiveness following PAE (Wong and Munro, 2014). Similarly, increased *Plvap* expression increases the breakdown and permeability of the blood-brain barrier (BBB; Shue et al., 2008). Given that ethanol increases the permeability of the BBB in adult mice, slight alterations in DNA methylation of *Plvap* could reflect broader effects on the BBB, which could, in turn, could affect downstream neurobiological functions (Alfonso-Loeches et al., 2016).

The tissue-concordant DMRs also contained several genes previously associated with mental health disorders. Although the same temporal stability could not be assessed here, as both tissues originated from the same age, these findings may point to more systemic effects of PAE on the developing organism. In particular, *Adh4* was differentially methylated across the hypothalamus and WBCs of PAE animals, and has been previously associated with alcohol dependence and substance abuse (Luo et al., 2005). Importantly, it is a key component of alcohol metabolism pathways, and could reflect increased susceptibility to the effects of alcohol during development. Furthermore, *Caln1* contained 3 separate DMRs; as it contains a risk allele for schizophrenia in some human populations, it could also play a role in some of the neuropsychiatric deficits observed in individuals with FASD (Li et al., 2015).

Of note, two genes displayed differential DNA methylation patterns in both the developmental profile and tissue-concordance analysis, *Cntnap5c* and *Ush2a*, which may reflect persistent alterations to DNA methylation patterns across both age and tissue types. In humans, genetic variation in *Cntnap5* is associated with risk for Alzheimer's disease and bipolar disorder, while its deletion is associated with autism and dyslexia, suggesting the possibility that common pathways may come into play among different neurobiological disorders (Pagnamenta et al., 2010; Xu et al., 2014; Schott et al., 2016). By contrast, mutations in *Ush2a* cause Usher syndrome II, which is associated with hearing deficiencies, deficits also commonly found in individuals with FASD (Church and Gerkin, 1988). Importantly, our animal model is based on an outbred population of Sprague-Dawley rats, which display fairly broad genetic diversity. Although genetic background can also influence DNA methylation patterns throughout the genome, the greater variability both within and between our treatment groups may reduce the likelihood of genetic variation having a major impact on our results and suggests that our results may represent more robust associations between PAE and DNA methylation patterns (Fraser et al., 2012; Heyn et al., 2013; Moen et al., 2013).

Finally, several DMRs in both datasets were located in genes associated with immune function and response. In particular, *Ifih1* was identified across all ages in the hypothalamus; as a receptor for double stranded RNA that responds to viral infections, it could be associated with vulnerability to immunological deficits (Rice et al., 2014). In addition, *Fgf9*, a key factor in embryonic and glial cell development, was differentially methylated in both the hypothalamus and WBCs (Thisse and Thisse, 2005). This growth factor promotes pro-inflammatory environments through Ccl2 and Ccl7 chemokine secretion, consistent with several DMRs that were located in genes associated with pro-inflammatory cytokine and chemokine signaling (Lindner et al., 2015). These included *Il20ra* and *Ccrl2* in the developmental profile, and *Il18r1* in the tissue-concordance analysis, suggesting that PAE could influence inflammatory pathways through epigenetic mechanisms, and ultimately, potentially alter brain development and the neuroimmune response.

We also assessed the functional enrichment of genes located within PAE-specific DMRs, identifying a number of biological processes associated with differential DNA methylation patterns in PAE animals compared to controls. In the DMRs identified across hypothalamic development, a large number of GO processes were associated with functions related to steroid receptor signaling. The hypothalamus is central to numerous physiological systems that function through steroid hormones, many of which are dysregulated by PAE (Weinberg et al., 2008). As such, this enrichment pattern suggests that DNA methylation may play a role in the reprogramming of hormonal systems during early development, potentially priming physiological systems to new set-points. In addition, several processes in both the developmental and tissue-concordant DMRs were associated with histone modifications, which may reflect the complex interplay between different layers of the epigenetic machinery. Several studies have identified alterations to histone modifications in the brain following developmental alcohol exposure, further highlighting their potential role in FASD (Guo et al., 2011; Govorko et al., 2012; Bekdash et al., 2013; Subbanna et al., 2013, 2014; Goldowitz et al., 2014; Veazey et al., 2015; Zhang et al., 2015; Chater-Diehl et al., 2016; Lussier et al., 2017). A large number of immune-related biological processes were also identified through this analysis, which is particularly relevant to individuals with FASD, who may be more susceptible to infections and immune deficits, a phenotype recapitulated across multiple animal models of PAE, and with some preliminary evidence in humans (Bodnar and Weinberg, 2013; Bodnar et al., 2016). Given the role of the hypothalamus in modulating the immune response, altered epigenetic programs could potentially contribute to altering baseline function and/or responsiveness to immune challenge of the hypothalamus, limiting the organism's ability to defend against disease or infection. In addition, the top GO term associated with PAE in the tissue-concordant DMRs was “cellular aldehyde metabolic process,” which may reflect lasting effects of PAE on the organism's ability to metabolize and tolerate alcohol's metabolic byproducts and possibly modulate susceptibility to substance abuse later in life. While no overlaps were identified between the specific biological processes identified in the developmental profile and tissue-concordance analyses, both contained a high proportion of processes associated with immune, endocrine, or epigenetic functions. These findings suggest that PAE may cause systemic effects on the epigenome across multiple tissue types, which may, in turn, influence downstream neurobiological and physiological processes.

Previous studies have identified subtle effects of PAE on gene expression programs and epigenomic patterns, which is consistent with the effects of other prenatal exposures (Rakyan et al., 2011; Zhou et al., 2011; Ladd-Acosta et al., 2013; Laufer et al., 2013; Berko et al., 2014; Lussier et al., 2015; Chater-Diehl et al., 2016). Regions containing lower CpG density appear to be more responsive to environmental exposures, highlighting the importance of selecting a method that covers a large portion of the epigenome when analyzing environmental exposures (Irizarry et al., 2009). We analyzed genome-wide DNA methylation using meDIP-seq, which reduces the complexity of the dataset by omitting unmethylated regions while interrogating a majority of genome (Harris et al., 2010). However, one limitation of meDIP-seq is that it is highly dependent on DNA methylation levels, local CpG density, and CpG position, which can introduce biases in the data (Pelizzola et al., 2008; Robinson et al., 2010b; Lentini et al., 2018). However, by performing pairwise comparisons between treatment and control groups, such biases are significantly reduced or eliminated (Harris et al., 2010). The enrichment patterns observed across our datasets are consistent with the fact that the majority of the mammalian genome is CpG-depleted and, with the exception of active regulatory regions, the remaining CpGs are methylated. By contrast, the vast majority of CpG-rich regions (i.e., promoters, CpG islands) are unmethylated and are less likely to bind to the 5-methylcytosine antibody, leading to depletion in meDIP-seq data, as observed in our own datasets. Consistent with the fact that promoter associated CpG islands are largely insensitive to environmental stimuli, few DMRs across our analyses were identified in promoters and CpG islands. Indeed, the majority of DMRs were located in intergenic regions and introns. Although these findings will require additional validation, these intergenic regions are critical to further explore, as they are potentially more responsive to the effects of PAE and may contain important regulatory regions not yet annotated in the rat genome. In addition, several DMRs were located in intron/exon boundaries. Given that DNA methylation plays a role in regulating alternative splice variants, these findings may reflect alterations to the balance of different isoforms within the cell, which could influence downstream cellular profiles and phenotypes (Maunakea et al., 2010, 2013; Shukla et al., 2011). Although isoform balance has not been investigated in the context of PAE, alcohol consumption can influence the proportions of different splice variants in the brain, supporting a potential role in early-life exposures as well (MacKay et al., 2011; Lee et al., 2014; Farris et al., 2015; Mathew et al., 2016). Interestingly, a larger proportion of down-methylated DMRs were identified in both analyses, which is consistent with several studies showing that PAE decreases bulk DNA methylation levels (Otero et al., 2012; Chen et al., 2013; Mukhopadhyay et al., 2013; Perkins et al., 2013; Liyanage et al., 2015; Nagre et al., 2015). These findings provide important insight into the various outcomes from different paradigms of alcohol exposure and suggest that similar upstream mechanisms may impact DNA methylation across models. In particular, alcohol is known to impact one-carbon metabolism through multiple pathways, including inhibition of folate-dependent methylation pathways and AdoMet synthesis (Medici and Halsted, 2013; Ngai et al., 2015). Furthermore, PAE can influence both the expression and activity of DNA methyltransferases, which are essential for the establishment and maintenance of DNA methylation profiles (reviewed in Lussier et al., 2017). These well-known mechanisms of alcohol-induced DNA methylation alterations likely contribute to the differential DNA methylation patterns observed across models and organisms, with specific effects potentially dictated by the timing and dosage of alcohol exposure, or other environmental factors (Pollard, 2007; Rogic et al., 2016; Lussier et al., 2017). For example, our animal model uses a constant level of alcohol exposure, resulting in blood alcohol levels of 80–150 mg/dl across the equivalent of the first two trimesters of human pregnancy. With this moderate to moderately high level of exposure, equivalent to 1–2 times the legal driving limit in most jurisdictions, we do not observe dysmorphologies or other severe growth and functional deficits. However, this paradigm does cause a broad range of behavioral and functional abnormalities, which are typically present across the entire spectrum of FASD. As such, the differential DNA methylation profiles observed in the present study potentially reflect a broader portion of the FASD spectrum in humans, and likely point to some of the common pathways affected by PAE across different exposure patterns.

The large proportion of DMRs located in intergenic regions suggests that these could contain regulatory regions susceptible to the influence of PAE. Given that the rat genome is poorly annotated for regulatory features, we assessed the enrichment profiles of different transcription factor binding sites in the DMRs, which could be influenced by DNA methylation levels within specific loci. While only the binding site for the BHLHE40 transcription factor was significantly enriched in PAE-specific DMRs across early development, we previously identified this gene as differentially expressed in the brain of PAE adult animals (Lussier et al., 2015). This gene negatively regulates the circadian rhythm, a key function of the hypothalamus that is dysregulated in individuals with FASD (Nakashima et al., 2008). The BHLHE40 transcription factor could potentially play a role in early programming effects of PAE on neurobiological systems, with persistent expression and downstream effect into later life. By contrast, the tissue-concordant DMRs contained a high proportion of significantly enriched TFBS, including SREBF1, which trended toward significance in the developmental profile DMRs. SREBF1 is associated with key metabolic processes for hormonal signaling, as it plays a role in the regulation of cholesterol production (Osborne, 2001). It is also associated with Smith–Magenis syndrome, which is characterized by intellectual disability, disordered sleep, and behavioral problems (Smith et al., 2002). Furthermore, additional TFBS enriched in the tissue-concordant dataset included two members of the forkhead box family of genes, including FOXK1 and FOXO3. In particular, FOXO3 was identified as a hub gene in the brain PAE animals following an immune challenge, suggesting that it may prime biological systems from early in life (Lussier et al., 2015). Finally, the highest represented TFBS in the tissue-concordant dataset was GMEB1, which is involved in signal transduction of the glucocorticoid response (Zeng et al., 2000). Importantly, recent evidence suggests that DNA methylation patterns can influence transcription factor occupancy, modulating the use of enhancer elements and gene expression levels (Maurano et al., 2015; Yin et al., 2017). Taken together, these findings suggest that the DMRs identified in both the developmental and tissue-concordant analysis may contain key regulatory regions, and that various transcription factors likely act in concert with DNA methylation to mediate the effects of PAE on physiological functions.

Although meDIP-seq allows for the investigation of more variable regions of the epigenome, it presents a particular caveat when assessing DNA methylation levels, as it provides relative levels of DNA methylation across broad regions of the genome, rather than quantitative and granular data. While a relative method was reasonable for the purpose of our study, which was to identify differences between animals exposed to alcohol and controls, we also attempted to verify our findings from the meDIP-seq analysis through bisulfite pyrosequencing, the gold standard for targeted DNA methylation analyses. However, the concordance between the two methods was not as strong as expected, with only the *Drd4* locus showing significant changes across the entire DMR. By contrast, other verified regions each showed a subset of CpGs that could be driving the associations. This discordance may potentially be due to a number of factors, including DNA hydroxymethylation, a bias of meDIP toward regions of high DNA methylation and CpG density, or sample size. A major limitation of the standard bisulfite pyrosequencing methods used here as a verification method for meDIP-seq is that it detects both methylated and hydroxymethylated cytosines, and there is no way to distinguish the two modifications using solely bisulfite conversion, resulting in a mixed signal. Although oxidative bisulfite conversion can distinguish DNA methylation and hydroxymethylation when used in parallel with bisulfite conversion, we could not perform this analysis on our samples due to DNA input limitations (Booth et al., 2012). By contrast, meDIP-seq specifically enriches DNA methylation, as the antibody is highly specific to 5-methylcytosine (Taiwo et al., 2012). Given that neuronal cells contain a high proportion of DNA hydroxymethylation compared to other cell types, it is possible that the observed differences in outcomes from the two methodologies are due to the confound of additional epigenetic patterns not assessed in the meDIP-seq analysis (Wang et al., 2012; Lister et al., 2013). Indeed, a number of studies have shown that developmental alcohol exposure can alter DNA hydroxymethylation programs in neuronal cells, suggesting that it may also play a role in the etiology of FASD and may have biased our verification analyses (Chen et al., 2013; Öztürk et al., 2017). In addition, the lack of verification could potentially be due to the relatively low number of animals used in the present study, as well as increased variability in the enrichment profiles obtained from meDIP-seq, given the broader regions assessed. Nevertheless, the *Drd4* locus identified in the developmental profile of the hypothalamus displayed consistent DNA methylation alterations in both methods, suggesting that meDIP-seq can indeed capture differences in DNA methylation patterns, regardless of the influence of DNA hydroxymethylation, and also highlighting the importance of the dopaminergic system in PAE-induced alterations. Additional studies are required to fully validate these findings and assess their relationship to the deficits observed following PAE. Given the high prevalence of DNA hydromethylation in the brain, future studies should endeavor to tease out the role of DNA methylation and DNA hydroxymethylation in the context of PAE, using methods that can quantitatively parse the two modifications (Wang et al., 2012; Lister et al., 2013).

A critical strength of animal models derives from their ability to directly compare central and peripheral tissues to ascertain potential correlations between the two, which may identify potential biomarkers reflective of brain function in a tissue that is available for study in human populations. Although several studies have assessed the concordance of DNA methylation patterns between different tissues in humans, the correlation between tissues tends to be rather low, highlighting the importance of analyzing both tissues in parallel (Farré et al., 2015; Edgar et al., 2017). Other than tissue identity, cell type heterogeneity within a tissue is a major driver of DNA methylation patterns. Therefore, we attempted to correct, at least partially, for cellular heterogeneity among groups by removing regions that were associated with the gene expression patterns of major cell types in the brain—neurons, astrocytes, and oligodendrocytes (Cahoy et al., 2008). However, we were limited by the use of regions located within genes, as well as the indirect usage of transcriptomes, and thus could not correct for important regulatory regions that may be associated with cell type or be poised for activation, such as intergenic enhancers or insulators. Furthermore, additional cellular subtypes, such as glia, are also present in the hypothalamus and could have influenced the results obtained through differences in cell-type proportions. Although we did not detect any significant PAE-specific differences in the DNA methylation levels of cell-type associated peaks, it is possible that the regions not captured by this peak-based filter could drive the differences in neuronal and glial function previously observed following PAE (Wilhelm and Guizzetti, 2015; Noor and Milligan, 2018). As several protocols now exist to isolate cell type-specific nuclei from both frozen and fresh tissue samples, future studies should attempt to replicate these findings in specific cellular subtypes to tease apart the role of DNA methylation in PAE-induced deficits (Habib et al., 2016; Milani et al., 2016).

By contrast, we measured the proportion of different WBC subtypes in an independent cohort of animals. The fact that we did not identify any significant differences in WBC composition of whole blood among groups suggests that blood composition does not drive the DMRs identified in the tissue-concordant analysis. There is also a small possibility that, as *Ficoll-Paque* is a highly technical procedure, differences between WBC extractions could have influenced the proportions of cells analyzed in the present study. Additionally, it is possible that group differences might be uncovered if cell subtypes are further subdivided through more sophisticated methods such as fluorescence-activated cell sorting. In contrast to human studies of DNA methylation, no bioinformatic tools exist to predict the proportion of different cell types using epigenomic profiles in rats, and future studies should take this into consideration. Nevertheless, we successfully identified several PAE-specific DMRs that showed the same direction of change between the two tissues, suggesting that these regions may be responsive to ethanol across multiple tissues and may represent more stable biomarkers of PAE.

Finally, female animals were the main focus of this epigenome-wide study, partially due to the broad sexual dimorphisms observed across studies of PAE and FASD (Lee and Rivier, 1996; Weinberg et al., 2008), as well as the widespread underrepresentation of females in neurobiological, molecular, and genome-wide studies of FASD (Lussier et al., 2017). However, as in the majority of studies utilizing only male subjects, this approach presents an important caveat in the interpretation of our results, as males and females often show sexually dimorphic responses to stress, disease, and other environmental factors, displaying differences in behavioral patterns, HPA axis function/activation, immune system activity, neurogenesis, and other physiological and cellular functions (Oldehinkel and Bouma, 2011; Bale and Epperson, 2015; Panzica and Melcangi, 2016; Yagi and Galea, 2018). In particular, the effects of PAE on the developing organism show marked sex-specific differences spanning cognitive and behavioral phenotypes, as well as in the differential susceptibilities to stressors and mental health disorders across the lifespan (Hellemans et al., 2008; Weinberg et al., 2008). Given that genetic and epigenetic patterns are highly associated with sex, research on females is vital to our understanding of the biological mechanisms underlying PAE (Zhang et al., 2011). Nevertheless, our findings should also be validated in male animals to fully assess the effects of PAE on the transcriptome and DNA methylome and elucidate the sexually dimorphic effects that may exist.

## Summary and Conclusions

Our results support and significantly extend previous studies indicating a role for DNA methylation in the early-life reprogramming of hypothalamic functions by PAE, and suggest that DNA methylation patterns in WBC could potentially be used as a surrogate for alterations in the central nervous system. We identified persistent PAE-induced alterations to the DNA methylome of the hypothalamus, including several DMRs that could, at least in part, underlie some of the deficits observed in FASD. Although PAE-induced alterations to DNA methylation profiles at any of these development ages may not persist into adulthood, changes early in development could alter developmental trajectories and induce lasting alterations in brain structure, connectivity, and function, and/or prime physiological systems to different set-points. Of note, we demonstrate for the first time that PAE-specific DMRs can occur concordantly across central and peripheral tissues, which potentially represent systemic effects of PAE on the epigenome and could serve as an epigenetic biomarker or signature of FASD. Taken together, these findings provide insight into the important role of epigenetic alterations in the short and long-term deficits observed in FASD and provide a foundation for the development of robust biomarkers of PAE.

## Data Availability Statement

The datasets generated for this study can be found in the Gene Expression Omnibus (GEO) database (GSE121582).

## Ethics Statement

All animal protocols were approved by the University of British Columbia Animal Care Committee and are consistent with the NIH Guide for the Care and Use of Laboratory Animals (certificates: A06-0017, A07-0381, A10-0136, A10-0016, A12-0032).

## Author Contributions

AL designed and performed all experiments and wrote the manuscript. TB helped with experimental design, data collection, and interpretations of results. MM helped with the meDIP-seq experiments and analyses. AM performed part of the pyrosequencing analyses. MH helped with design and analysis of meDIP-seq data. MK and JW contributed to all stages, including design, analysis, interpretation, and writing.

### Conflict of Interest Statement

The authors declare that the research was conducted in the absence of any commercial or financial relationships that could be construed as a potential conflict of interest.
