# Supplementary material for: Prenatal Alcohol Exposure: Profiling Developmental DNA Methylation Patterns in Central and Peripheral Tissues
Source: Front Genet. 2018 Dec 4;9:610. doi: 10.3389/fgene.2018.00610 (PMC6290329; doi:10.3389/fgene.2018.00610)
Supplement: Supplementary file 2 [file Data_Sheet_1.PDF]

Supplementary Material

Prenatal alcohol exposure: profiling developmental DNA methylation patterns in central and peripheral tissues

Lussier AA[1,2,3*], Bodnar TS[2], Mingay M[4], Morin AM[1], Hirst M[4,5], Kobor MS[2,6§], Weinberg J[3§]

*** Correspondance:** Alexandre Lussier: alussier@cmmt.ubc.ca

1 Supplementary Data

1.1 Statistical analyses of developmental data

Maternal data during gestation and lactation were analyzed using repeated measures analyses of variance (ANOVA), with prenatal treatment as the between-subjects factor, and postnatal day as the within-subjects factor. As separate cohorts of offspring from each prenatal group were terminated on P1, P8, P15, or P22 (n = 4/group/age/tissue), body weights were analyzed by ANOVAs for the factor of prenatal treatment at each age and in a group*age interaction model. Blood composition data were also analyzed using a two-way ANOVA to identify differences among groups for each WBC subtype. Significant main effects and interactions were further analyzed by Tukey honest significant difference (HSD) *post hoc* tests (p<0.05).

On average, alcohol intake of PAE dams was consistently high across pregnancy, ranging from 0.208 ± 0.014 to 0.268 ± 0.022 mL/kg body weight during *week 1*, 0.240 ± 0.016 to 0.305 ± 0.017 during *week 2*, and 0.236 ±0.014 to 0.285 ± 0.019 during *week 3* of gestation. These levels of drinking typically result in blood alcohol levels ~100-150 mg/dL [54,55]. Analysis of maternal body weights during gestation (GD1, 7, 14, 21) and following parturition (P1, 8, 15, 22) showed significant main effects of group ($F_{(2,143)}$=13.609, p = 3.9 e-6 and $F_{(2,91)}$=9.559, p =1.7 e-4, respectively) and group X day interactions ($F_{(6,143)}$ = 2.869, p = 0.011 and $F_{(2,91)}$ = 2.566, p = 0.082, respectively) during both gestation and lactation. Both PAE and PF dams weighed significantly less than controls on GD14 and 21, and following parturition (P1). However, catch-up weight gain occurred after birth and maternal weight differences among groups were no longer significant by P8.

We did not observe any significant group differences in the number of live-born pups, or in the average weight of female pups/litter at any of the collection days (Supplementary Table 1)

2 Supplementary Figures

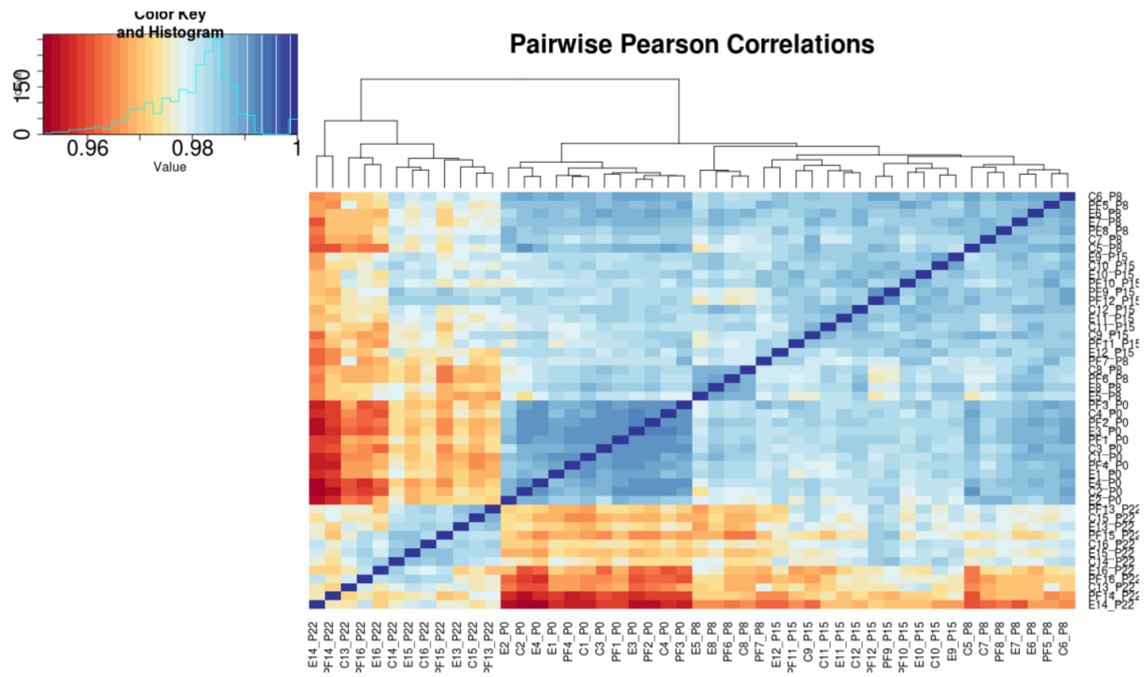

Supplementary figure 1. Pairwise Pearson correlations of meDIP-seq data for the developmental hypothalamus samples. Samples were generally highly correlated (r<0.95), with samples clustering most closely with animals of the same age.

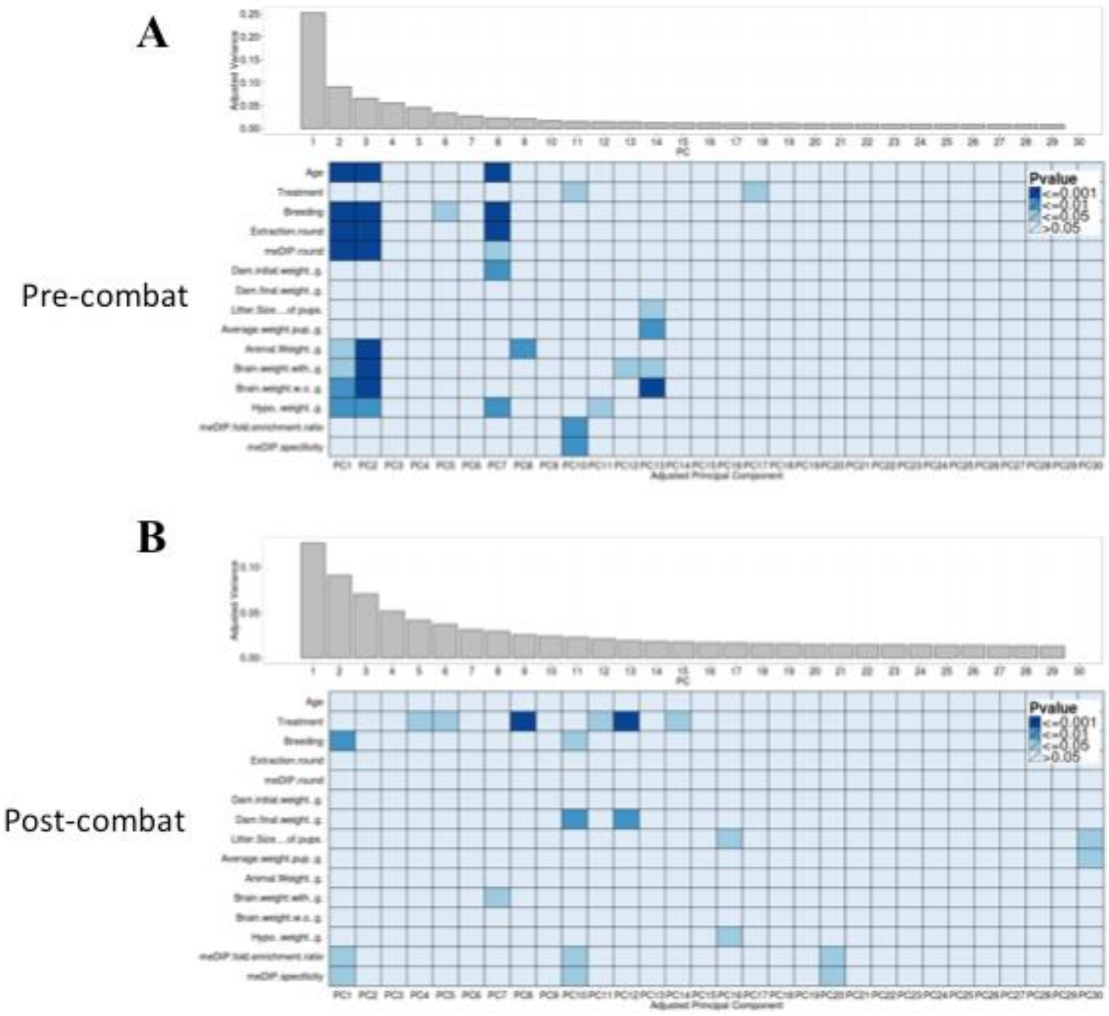

Supplementary figure 2. Principal component analysis of meDIP-seq data for the hypothalamus samples before and after ComBat correction. A) Principal component analysis of the normalized RPKM data revealed significant levels of variation associated with batch effects. MeDIP and DNA extraction rounds were significantly associated with a large proportion of variation within the dataset, and were also confounded with age. **B)** ComBat correction was performed on the RPKM data from the hypothalamic samples to correct the effects of MeDIP round and DNA extraction round. Partial effects of breeding remained in the dataset and prenatal treatment was associated with a larger proportion of variance within the dataset after ComBat correction.

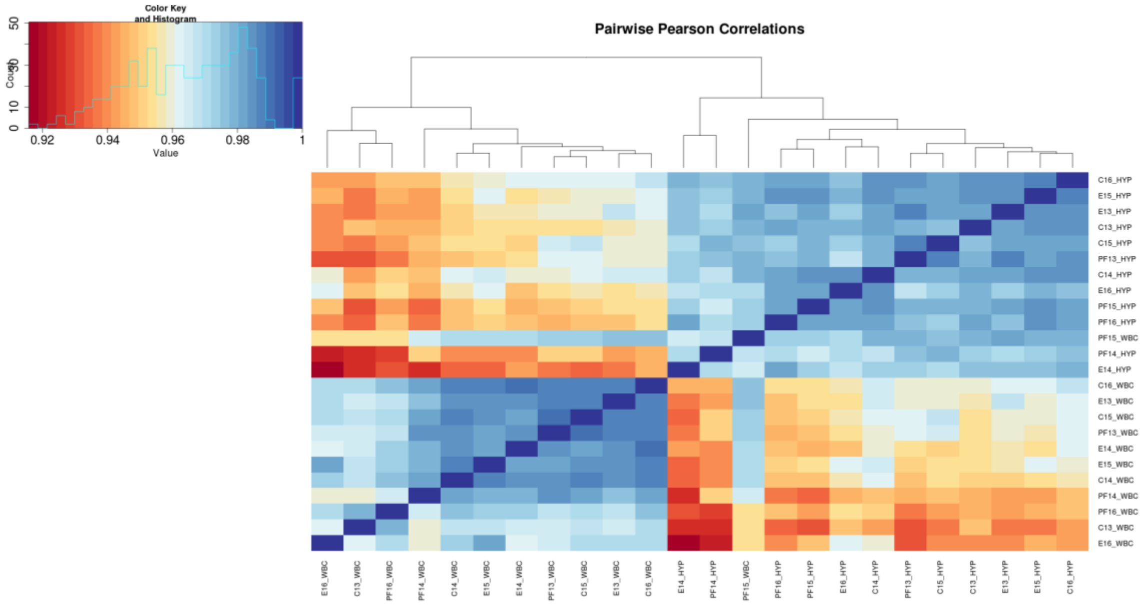

Supplementary figure 3. Pairwise Pearson correlations of meDIP-seq data for the hypothalamus and white blood cell samples on postnatal day 22. Samples in the BvB peakset were highly correlated within tissue (r>0.96), the main driver of DNA methylation patterns, and well correlated within the same animals (r>0.92). However, one PF WBC sample clustered with the hypothalamus samples, suggesting that it may have been mislabeled during processing.

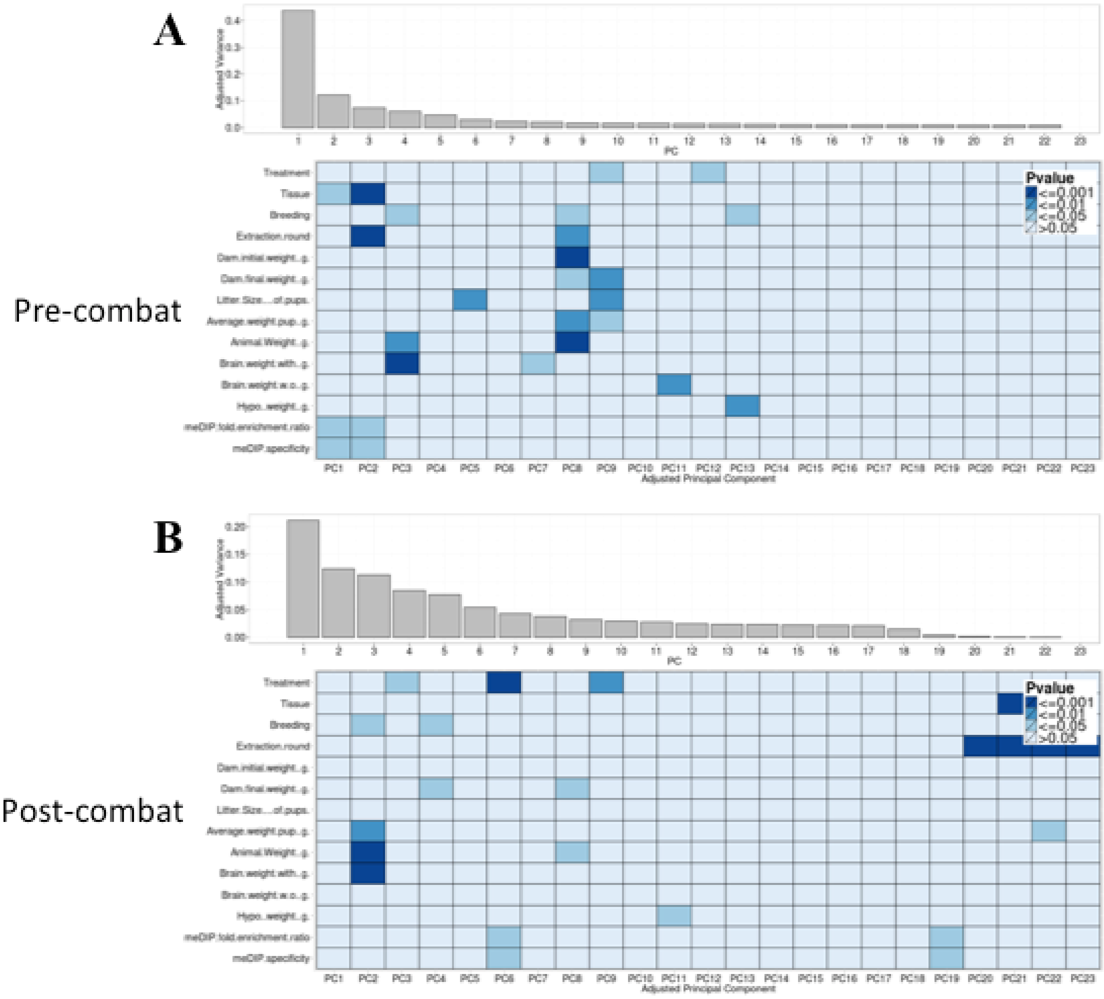

Supplementary figure 4. Principal component analysis of meDIP-seq data for the postnatal day 22 hypothalamus and white blood cell samples before and after ComBat correction. A) Principal component analysis of the normalized BvB RPKM data revealed significant levels of variation associated with DNA extraction round batch effects**.** Tissue type was the covariate most strongly associated with variance in the dataset, although it was slightly confounded with extraction round. B) ComBat correction was used to account for the effects of DNA extraction round in the BvB dataset, though this also removed cell-type associated variation. Prenatal treatment was associated with a larger proportion of variance within the dataset following ComBat correction.

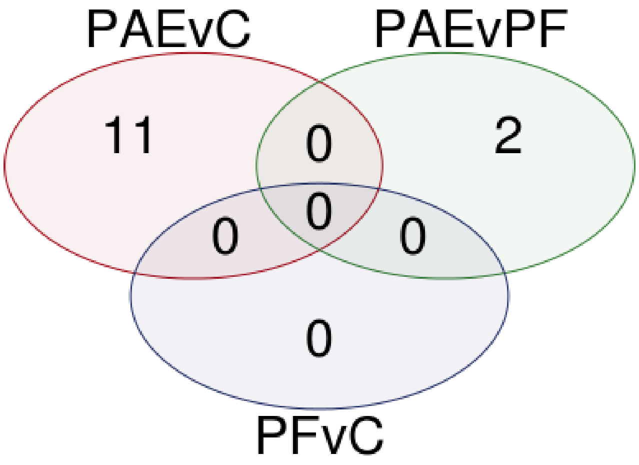

Supplementary figure 5. Cell-type associated DMRs. Linear modeling was performed on the 18,050 peaks located in cell-type associated meDIP-seq peaks, correcting for age and breeding. At an FDR<0.05, 11 DMRs were identified between PAE and controls (C; 6 neuron-, 3 oligodendrocyte-, 2 astrocyte-related), while 2 DMRs were found between PAE and PF (1 neuron-, 1 oligodendrocyte-related). No DMRs were identified between PF and controls, and no overlaps were identified.

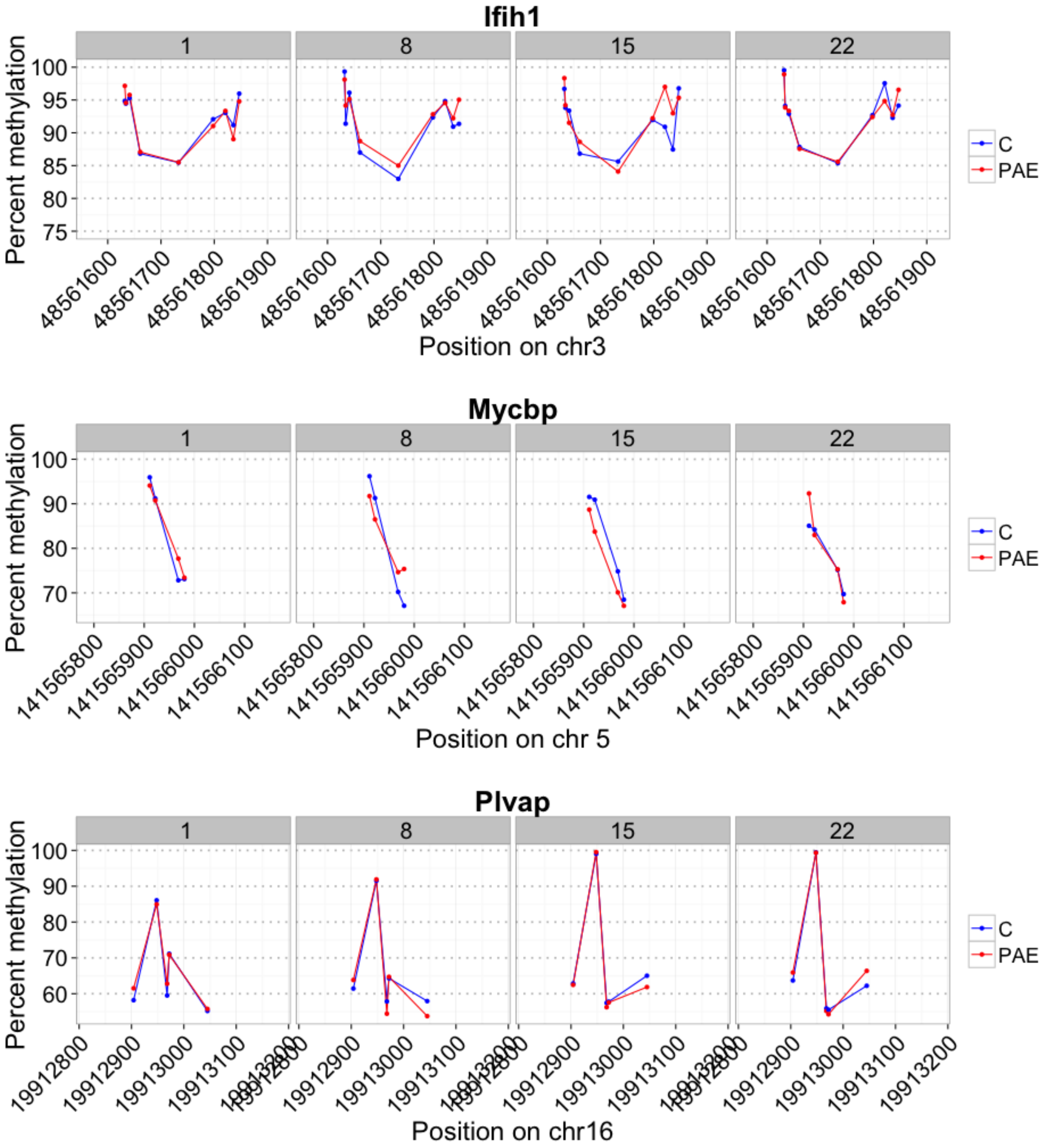

Supplementary figure 6. Additional bisulfite pyrosequencing data for the developmental hypothalamus samples. Three additional DMRs were verified by bisulfite pyrosequencing using the hypothamaus samples at P1, P8, P15, and P22. These were located within *Ifih1* (chr3:48,561,559-48,561,925), *Mycbp* (chr5:141,565,784-141,566,172), and *Plvap* (chr16:19,912,813-19,913,185). PAE = red; Control = blue.

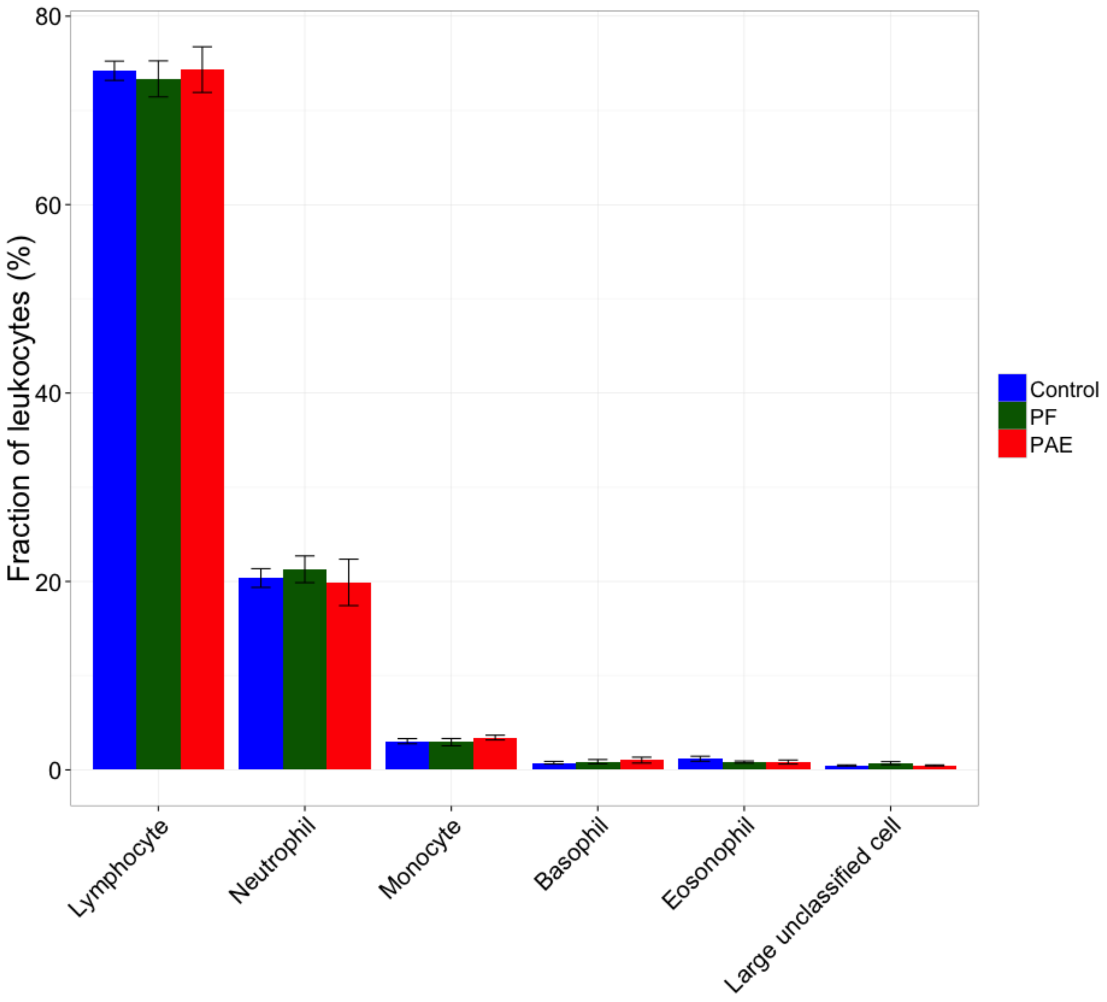

Supplementary figure 7. White blood cell composition on postnatal day 22. The composition of white blood cells from PAE, PF, and C animals on P22 was analyzed to obtain the proportions of lymphocytes, neutrophils, monocytes, basophils, eosoniphils, and large unclassified cells. No significant differences were observed between groups.

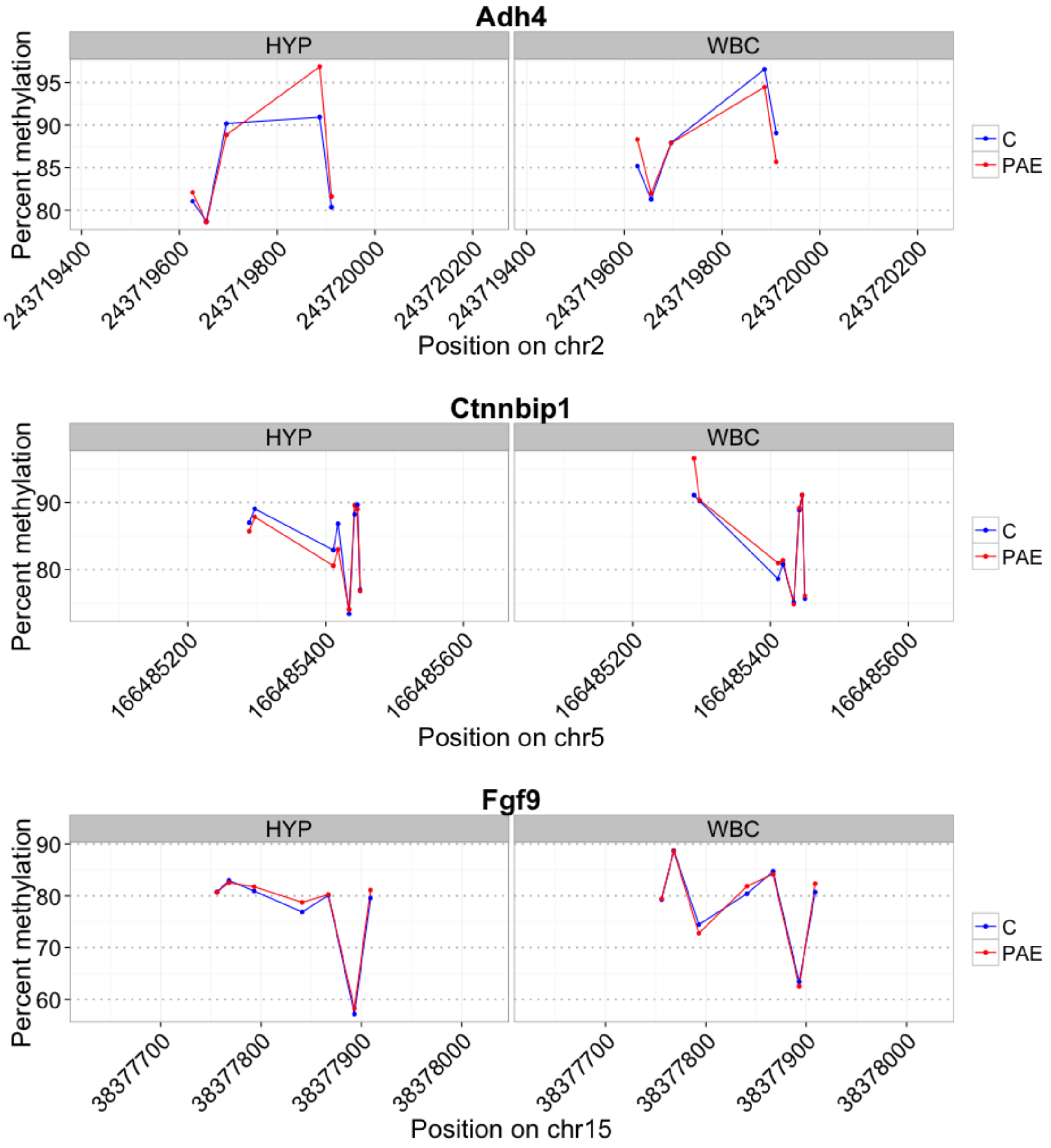

Supplementary figure 8. Bisulfite pyrosequencing data for the postnatal day 22 hypothalamus and white blood cell samples. DNA methylation patterns were analyzed in the final exon and 3' UTR of *Adh4* (chr2: 243,719,416-243,720,233), the first exon and 5' UTR of *Ctnnbip1* (chr5: 166,485,057-166,485,637), and the first intron of *Ffg9* (chr15: 38,377,629-38,378,027). PAE = red, Control = blue.
